# Supplementary material for: A large and diverse autosomal haplotype is associated with sex-linked colour polymorphism in the guppy
Source: Nat Commun. 2022 Mar 9;13:1233. doi: 10.1038/s41467-022-28895-4 (PMC8907176; doi:10.1038/s41467-022-28895-4)
Supplement: Supplementary file 1 — Supplementary Information [file 41467_2022_28895_MOESM1_ESM.pdf]

Supplementary Information

A large and diverse autosomal haplotype is associated with sex-linked colour polymorphism in the guppy

Josephine R Paris, James R Whiting, Mitchel J Daniel, Joan Ferrer Obiol, Paul J Parsons,

Mijke J van der Zee, Christopher W Wheat, Kimberly A Hughes and Bonnie A Fraser

This PDF includes:

Supplementary Table 1: Pseudo-F, R^2 and P values from pairwise permutational MANOVA

Supplementary Table 2: Phenotypic variances and P-values from permutational t-tests comparing variances

Supplementary Table 3: Loadings of Z-F_{ST} principal components for whole genome

Supplementary Table 4: Loadings of Z-F_{ST} principal components for LG1

Supplementary Table 5: Raw pairwise F_{ST} between Iso-Y lines (per-SNP)

Supplementary Table 6: Loadings of Z-F_{ST} principal components for LG12

Supplementary Table 7: Loadings of Z-π principal components on LG1

Supplementary Table 8: Loadings of Z-π principal components on LG12

Supplementary Table 9: Sequencing information for the Iso-Y lines.

Supplementary Figure 1: Discriminant analysis of principal components (DAPC) DF2 & DF3

Supplementary Figure 2: Density plots for the first 17 principal components

Supplementary Figure 3: Observed and permuted differences in phenotypic variance

Supplementary Figure 4: Percentage of SNPs with a high Z-F_{ST} PC1 above the upper 95% quantile

Supplementary Figure 5: Pairwise F_{ST} and recombination between the four Iso-Y lines on other autosomes

Supplementary Figure 6: Assessment of the relationship between recombination and F_{ST} differentiation

Supplementary Figure 7: LG1 Pairwise F_{ST} calculated between the four Iso-Y lines

Supplementary Figure 8: Z-F_{ST} PC2 for LG1

Supplementary Figure 9: LG1 Z- F_{ST} PC1 and recombination landscape

Supplementary Figure 10: LG12 Pairwise F_{ST} calculated between the four Iso-Y lines

Supplementary Figure 11: Z-F_{ST} PC2 and Z-F_{ST} PC3 for LG12

Supplementary Figure 12: Z-π-LG1 PC1 (74% of variance) along LG1

Supplementary Figure 13: Coverage calculated across LG1 for each of the four Iso-Y lines

Supplementary Figure 14: Z-π PC1 and Z-π PC2 along LG12

Supplementary Figure 15: LG1 polarised allele frequencies

Supplementary Figure 16: Allele frequency (AF) density distributions for LG1 Region 2

Supplementary Figure 17: Allele frequency (AF) density distributions for LG1 Region 3

Supplementary Figure 18: Patterns of linkage disequilibrium (LD) for males and females

Supplementary Figure 19: GC content of LG1 and LG12 in 1kb windows

Supplementary Figure 20: Repeat content of LG1 and LG12 in 1kb windows

Supplementary Figure 21: SNP density of LG1 in the natural data
Supplementary Figure 22: Coverage calculated across LG1 for the natural data
Supplementary Figure 23: Gene density of LG1 from the male guppy genome
Supplementary Figure 24: *Lostruct* local PCA analysis for LG1 and LG12
Supplementary Figure 25: Inter-chromosomal linkage between LG1 and LG12
Supplementary Figure 26: Comparisons of LG1 among natural HP-LP populations
Supplementary References

Supplementary Table 1. Pseudo-F, R^2 and P values from pairwise permutational MANOVA (below the diagonal) and distances between centroids (above the diagonal) for Iso-Y line phenotype.

	Iso-Y6	***Iso-Y8***	***Iso-Y9***	***Iso-Y10***
Iso-Y6		43.19	70.861	81.397
Iso-Y8	pseudo-F1,88 = 12.861, R^2 = 0.129, P < 0.001		67.055	70.638
Iso-Y9	pseudo-F1,81 = 18.446, R^2 = 0.185, P < 0.001	pseudo-F1,88 = 23.07, R^2 = 0.208, P < 0.001		72.606
Iso-Y10	pseudo-F1,81 = 33.551, R^2 = 0.293, P < 0.001	pseudo-F1,88 = 37.769, R^2 = 0.300, P < 0.001	pseudo-F1,82 = 20.507, R^2 = 0.2, P < 0.001	

Supplementary Table 2. Phenotypic variances (diagonal) and P-values from permutational t-tests comparing variances (off-diagonal) between Iso-Y lines.

	Iso-Y6	***Iso-Y8***	***Iso-Y9***	***Iso-Y10***
Iso-Y6	29.359			
Iso-Y8	*0.013*	21.959		
Iso-Y9	*0.002*	*<0.001*	37.987	
Iso-Y10	0.791	*0.015*	*<0.001*	28.575

Supplementary Table 3. Loadings of Z-F_{ST} principal components on the six different pairwise comparisons across the whole genome. PC1 (37% of total variance) showed high positive loadings of all pairwise comparisons. Proportion of variances for remaining PCs: PC2 (20%); PC3 (17%); PC4 (12%); PC5 (8%); PC6 (6%).

Comparison	***PC1***	***PC2***	***PC3***	***PC4***	***PC5***	***PC6***
Iso-Y10 vs Iso-Y6	0.40	-0.50	0.30	-0.29	0.54	-0.35
Iso-Y10 vs Iso-Y8	0.31	-0.11	-0.81	-0.14	0.28	0.37
Iso-Y10 vs Iso-Y9	0.38	0.51	0.29	0.45	0.47	0.31
Iso-Y6 vs Iso-Y8	0.31	-0.59	0.02	0.65	-0.35	0.13
Iso-Y6 vs Iso-Y9	0.50	0.08	0.30	-0.51	-0.46	0.43
Iso-Y8 vs Iso-Y9	0.51	0.37	-0.27	0.08	-0.28	-0.67

Supplementary Table 4. Loadings of Z-F_{ST} principal components on the six different pairwise comparisons on LG1. PC1 (52% of total variance) showed high positive loadings of all pairwise comparisons except for Iso-Y6 vs Iso-Y8, which loaded strongly onto PC2 (PC2 captured 17% of variance). Other proportion of variances for remaining PCs: PC3 (16%). PC4 (11%), PC5 (2%). PC6 (0%).

Comparison	***PC1***	***PC2***	***PC3***	***PC4***	***PC5***	***PC6***
Iso-Y10 vs Iso-Y6	0.42	0.17	-0.04	0.76	-0.23	-0.40
Iso-Y10 vs Iso-Y8	0.36	0.35	-0.61	-0.35	-0.47	0.20
Iso-Y10 vs Iso-Y9	0.38	-0.47	0.45	-0.37	-0.47	-0.28
Iso-Y6 vs Iso-Y8	0.04	-0.77	-0.61	0.13	0.13	-0.05
Iso-Y6 vs Iso-Y9	0.53	-0.14	0.22	0.20	0.17	0.76
Iso-Y8 vs Iso-Y9	0.52	0.16	-0.06	-0.32	0.68	-0.38

Supplementary Table 5. Raw pairwise F_{ST} between Iso-Y lines (per-SNP).

	Iso-Y10 vs Iso-Y6	*Iso-Y10 vs Iso-Y8*	*Iso-Y10 vs Iso-Y9*	*Iso-Y6 vs Iso-Y8*	*Iso-Y6 vs Iso-Y9*	*Iso-Y8 vs Iso-Y9*
LG1	0.11	0.11	0.09	0.05	0.26	0.24
LG2	0.07	0.06	0.04	0.11	0.09	0.03
LG3	0.05	0.09	0.06	0.12	0.05	0.13
LG4	0.07	0.09	0.05	0.11	0.10	0.08
LG5	0.05	0.06	0.04	0.05	0.09	0.10
LG6	0.09	0.03	0.06	0.07	0.11	0.09
LG7	0.13	0.07	0.08	0.07	0.07	0.06
LG8	0.09	0.06	0.05	0.07	0.15	0.15
LG9	0.06	0.06	0.04	0.09	0.03	0.08
LG10	0.09	0.04	0.04	0.08	0.05	0.04
LG11	0.08	0.04	0.06	0.07	0.16	0.10
LG12	0.12	0.04	0.21	0.13	0.22	0.20
LG13	0.05	0.05	0.03	0.07	0.06	0.08
LG14	0.09	0.05	0.08	0.05	0.16	0.13
LG15	0.05	0.04	0.09	0.08	0.06	0.10
LG16	0.06	0.06	0.03	0.03	0.06	0.06
LG17	0.06	0.03	0.03	0.04	0.08	0.04
LG18	0.08	0.07	0.03	0.08	0.08	0.09
LG19	0.11	0.02	0.06	0.11	0.16	0.08
LG20	0.07	0.05	0.09	0.07	0.10	0.07
LG21	0.14	0.07	0.04	0.09	0.12	0.09
LG22	0.12	0.11	0.08	0.19	0.08	0.14
LG23	0.08	0.05	0.04	0.06	0.09	0.07

Supplementary Table 6. Loadings of Z-F_{ST} principal components on the six different pairwise comparisons on LG12. PC1 (37% of total variance) showed positive loadings of all pairwise comparisons except for Iso-Y6 vs Iso-Y8, which loaded onto PC2 (30%). Other proportion of variances for remaining PCs: PC3 (16%); PC4 (10%); PC5 (4%); PC6 (3%).

Comparison	***PC1***	***PC2***	***PC3***	***PC4***	***PC5***	***PC6***
Iso-Y10 vs Iso-Y6	0.18	-0.66	0.20	-0.07	0.56	-0.43
Iso-Y10 vs Iso-Y8	0.04	-0.23	-0.96	0.13	0.07	-0.02
Iso-Y10 vs Iso-Y9	0.59	0.17	0.01	0.32	-0.41	-0.59
Iso-Y6 vs Iso-Y8	0.19	-0.66	0.11	-0.06	-0.60	0.39
Iso-Y6 vs Iso-Y9	0.47	0.18	-0.14	-0.85	0.04	0.05
Iso-Y8 vs Iso-Y9	0.59	0.12	0.07	0.39	0.40	0.56

Supplementary Table 7. Loadings of Z-π principal components on the four Iso-Y lines on LG1. PC1 (74% of total variance) showed positive loadings of Iso-Y lines. Other proportion of variances for remaining PCs: PC2 (21%); PC3 (3%); PC4 (1%).

	PC1	***PC2***	***PC3***	***PC4***
Iso-Y10	0.55	-0.02	-0.83	0.10
Iso-Y9	0.30	-0.92	0.21	-0.14
Iso-Y8	0.56	0.19	0.45	0.67
Iso-Y6	0.54	0.34	0.26	-0.72

Supplementary Table 8. Loadings of Z-π principal components on the four Iso-Y lines on LG12. PC1 (88% of total variance) showed positive loadings of Iso-Y lines. Other proportion of variances for remaining PCs: PC2 (7%); PC3 (3%); PC4 (2%).

	PC1	***PC2***	***PC3***	***PC4***
Iso-Y10	0.51	-0.26	0.16	-0.80
Iso-Y9	0.47	0.87	-0.12	-0.01
Iso-Y8	0.51	-0.18	0.66	0.52
Iso-Y6	0.50	-0.37	-0.72	0.30

Supplementary Table 9. Sequencing information for the Pool-seq data generated for each of the Iso-Y lines.

	Raw reads	***Clean reads***	***Paired & mapped***	***Alignment rate***	***Coverage***
Iso-Y6	731,979,270	723,283,568	723,283,568	0.9615	118X
Iso-Y8	809,452,924	800,743,504	770,402,246	0.9621	131X
Iso-Y9	779,997,590	768,182,786	739,052,904	0.9621	126X
Iso-Y10	751,971,468	740,921,674	712,267,486	0.9613	121X

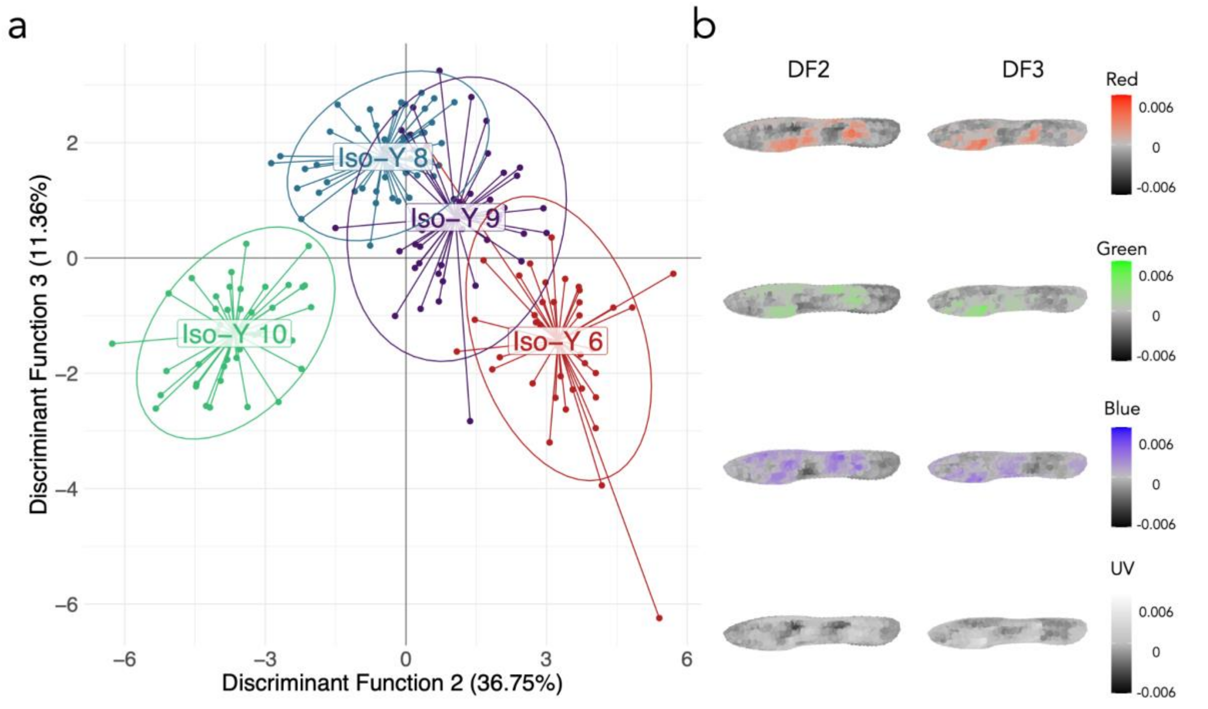

Supplementary Figure 1. Discriminant analysis of principal components (DAPC). (a) Scatterplot of discriminant functions 2 & 3. (b) Heatmaps for each colour channel depicting the correlations between colour and at each sampling location and discriminant function 2 or 3. Source data underlying Supplementary Figure 1a are provided as a Source Data file.

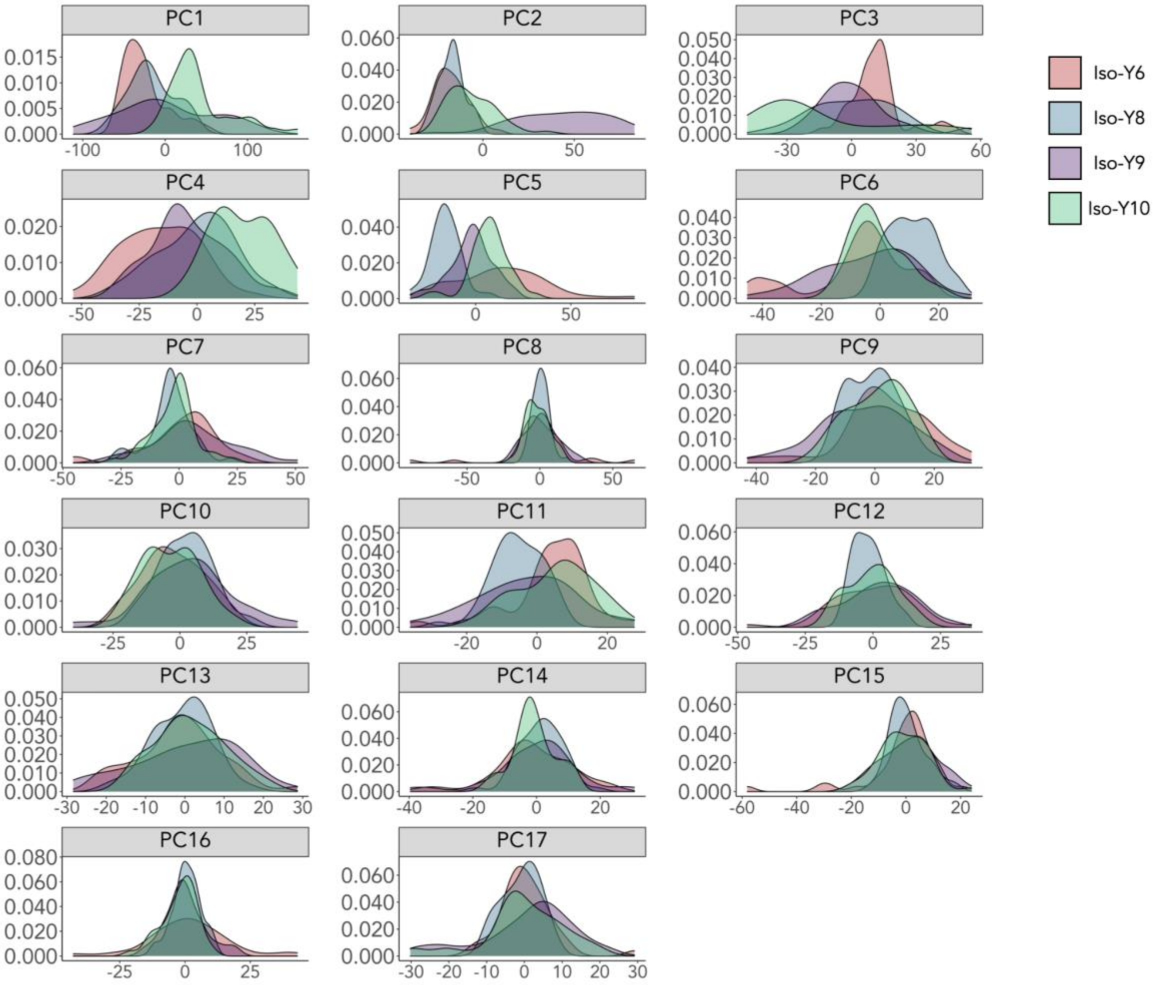

Supplementary Figure 2. Density plots of the phenotype of each Iso-Y line for the first 17 principal components.

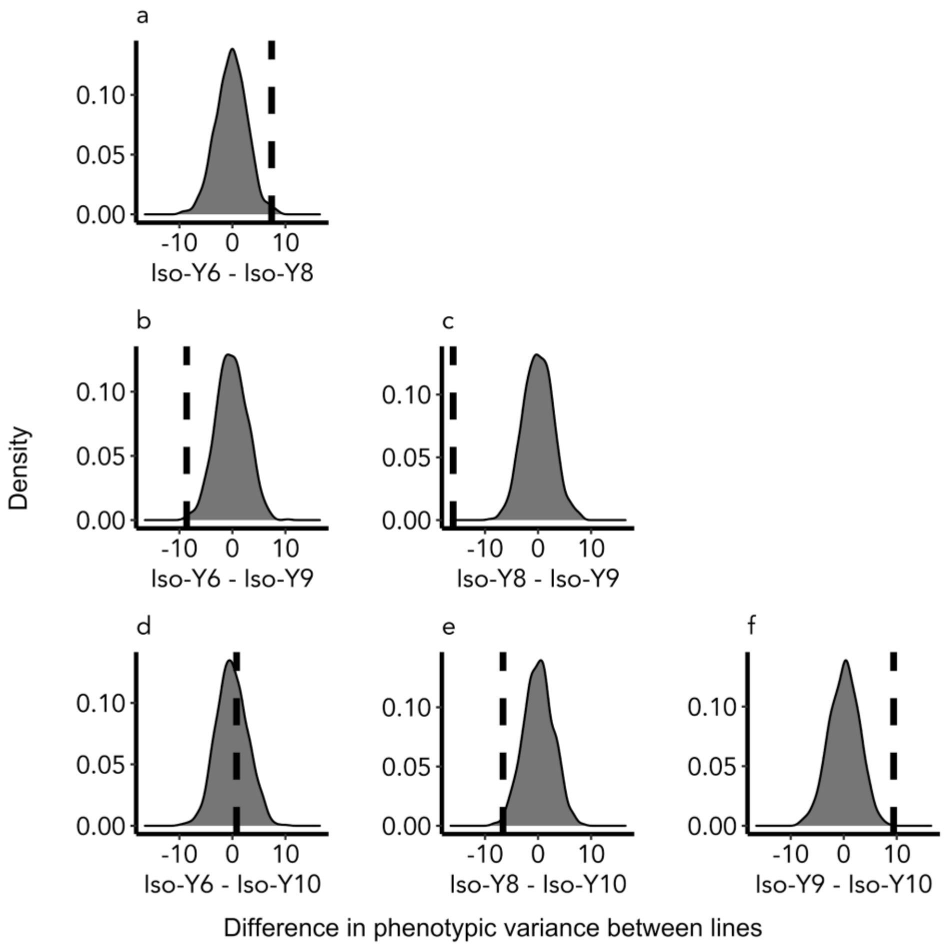

Supplementary Figure 3. Observed and permuted differences in phenotypic variance between each pair of Iso-Y lines. The density curve describes the null distribution of variances; the dashed line denotes the observed difference in variance.

a

b

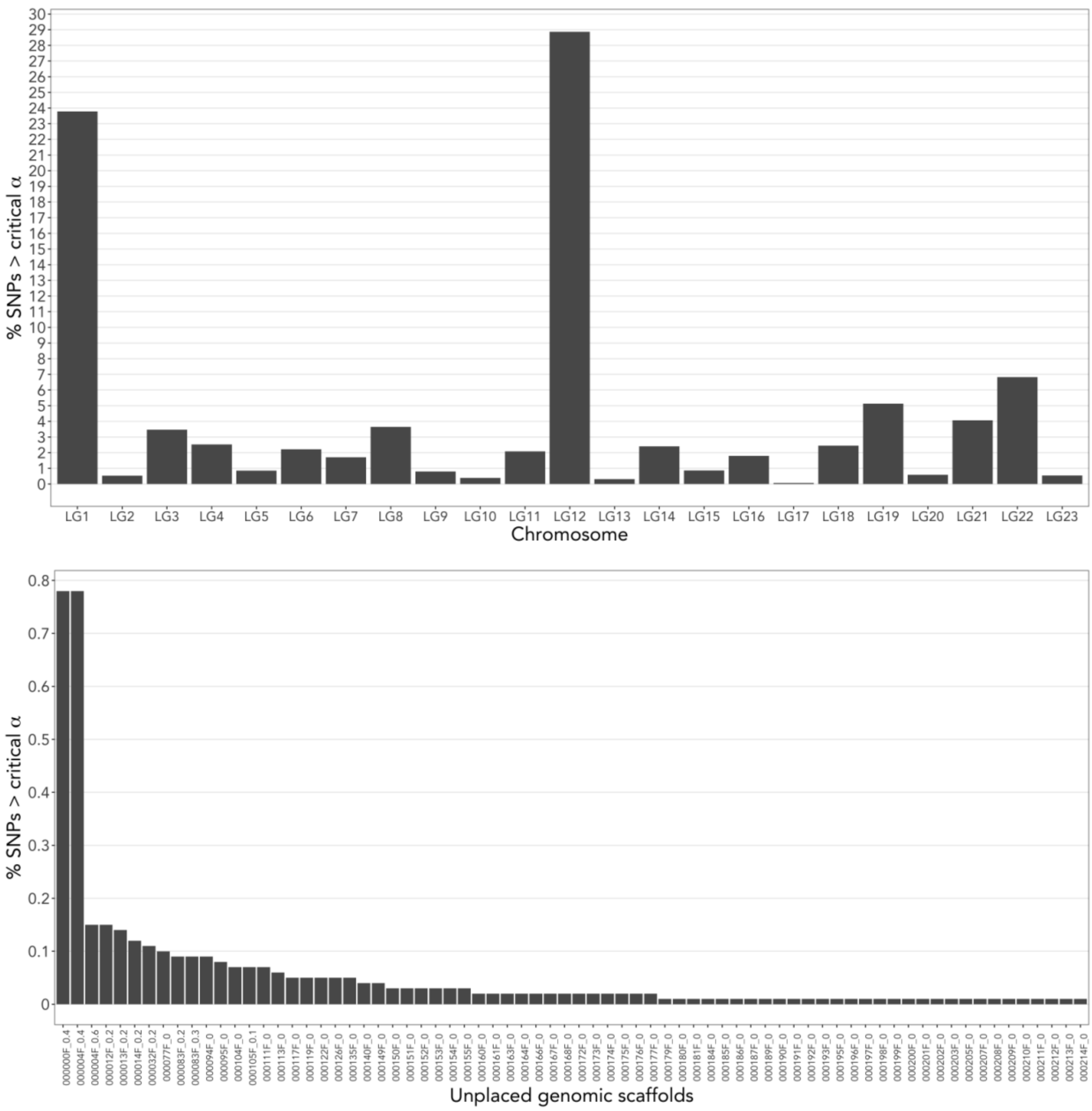

Supplementary Figure 4. Percentage of SNPs with a high Z-F_{ST} PC1 above the upper 95% quantile (Z-F_{ST} PC1 > 3) across the genome: a) per chromosome; b) per unplaced genomic scaffold (showing only scaffolds which showed high-scoring SNPs).

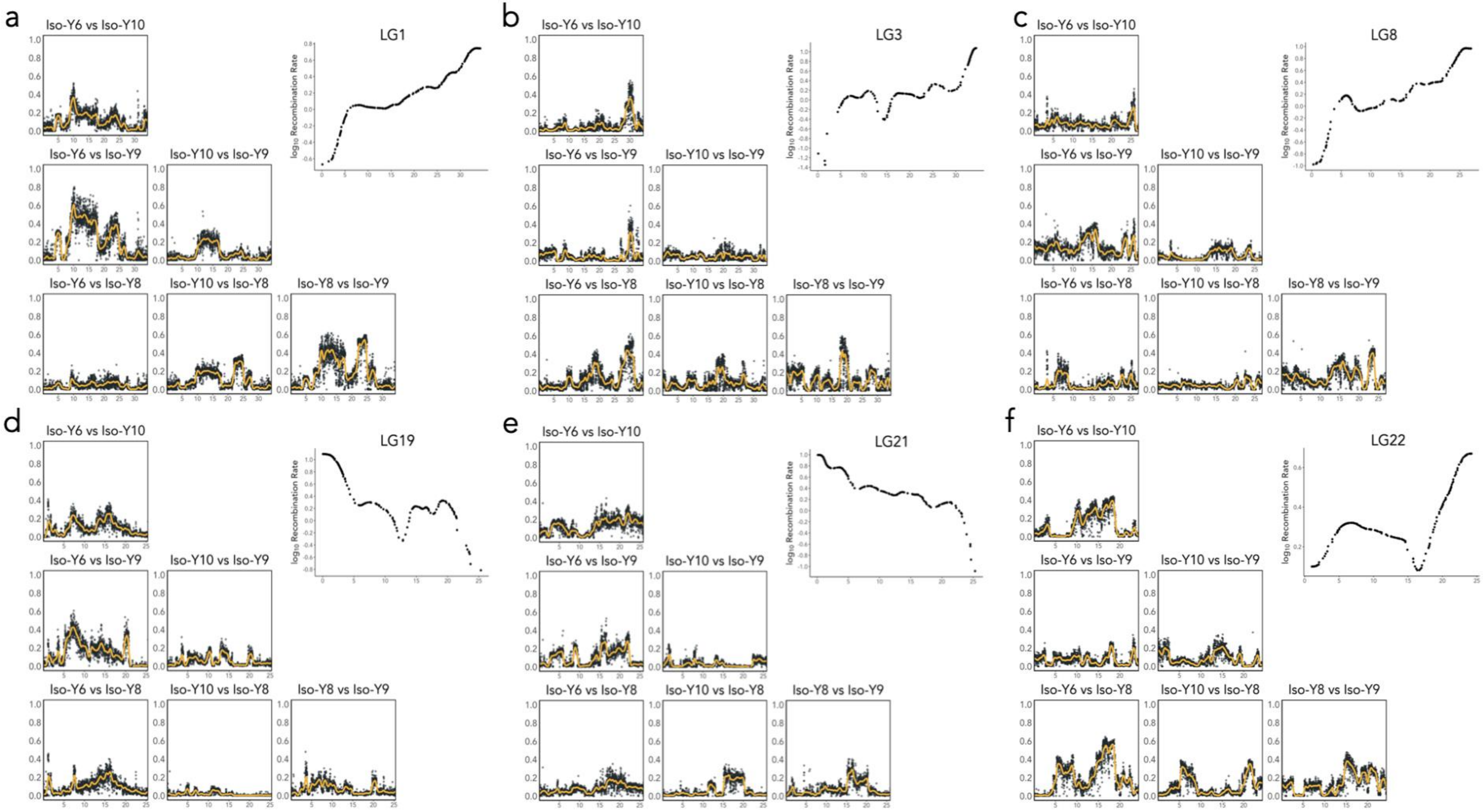

Supplementary Figure 5. (see next page for legend).

Supplementary Figure 5. Pairwise F_{ST} calculated in 10kb windows between the four Iso-Y lines on autosomes with high Z-F_{ST} PC1-scoring SNPs above the upper 95% quantile. Yellow lines represent a smoothed spline of the data. Adjacent to each panel, smoothed recombination rate is shown. Recombination data were derived from four independent F2 QTL crosses [1] through personal communication with the authors who provided permissions. The linkage map consists of 6,765 markers and a length of 1,673.8 cM. To extract information on recombination rate, maps were smoothed using the *smooth.spline* function in R. Source data for these maps are provided as a Source Data file. Smoothing was necessary to estimate recombination over regions where individual markers were not syntenic with the published reference genome (micro-rearrangements). Larger rearrangements (such as assembly errors and inversions), where multiple markers were out of order, were manually re-oriented resulting in smoothed maps. Smoothed maps have an average of 242 markers per chromosome. These recombination data are sparse, and so likely underestimate recombination coldspots/hotspots. However, the maps represent the highest-density markers available for the species. Moreover, they capture the broad acrocentric nature of recombination in the guppy chromosomes [2,3]. (a) LG1 (24% of high-scoring SNPs) is included as a comparison to other autosomes which showed proportions >3% of high-scoring SNPs (see Supplementary Fig. 5): (b) LG3 (3.5% of high-scoring SNPs); (c) LG8 (3.65% of high-scoring SNPs); (d) LG19 (5.1% of high-scoring SNPs); (e) LG21 (4% of high-scoring SNPs); (f) LG22 (6.8% of high-scoring SNPs). It is clear from the pairwise comparisons that high-scoring SNPs on other autosomes represent differentiation unique to a particular Iso-Y line. On LG22 (f), some differentiation is associated with a suspected recombination coldspot.

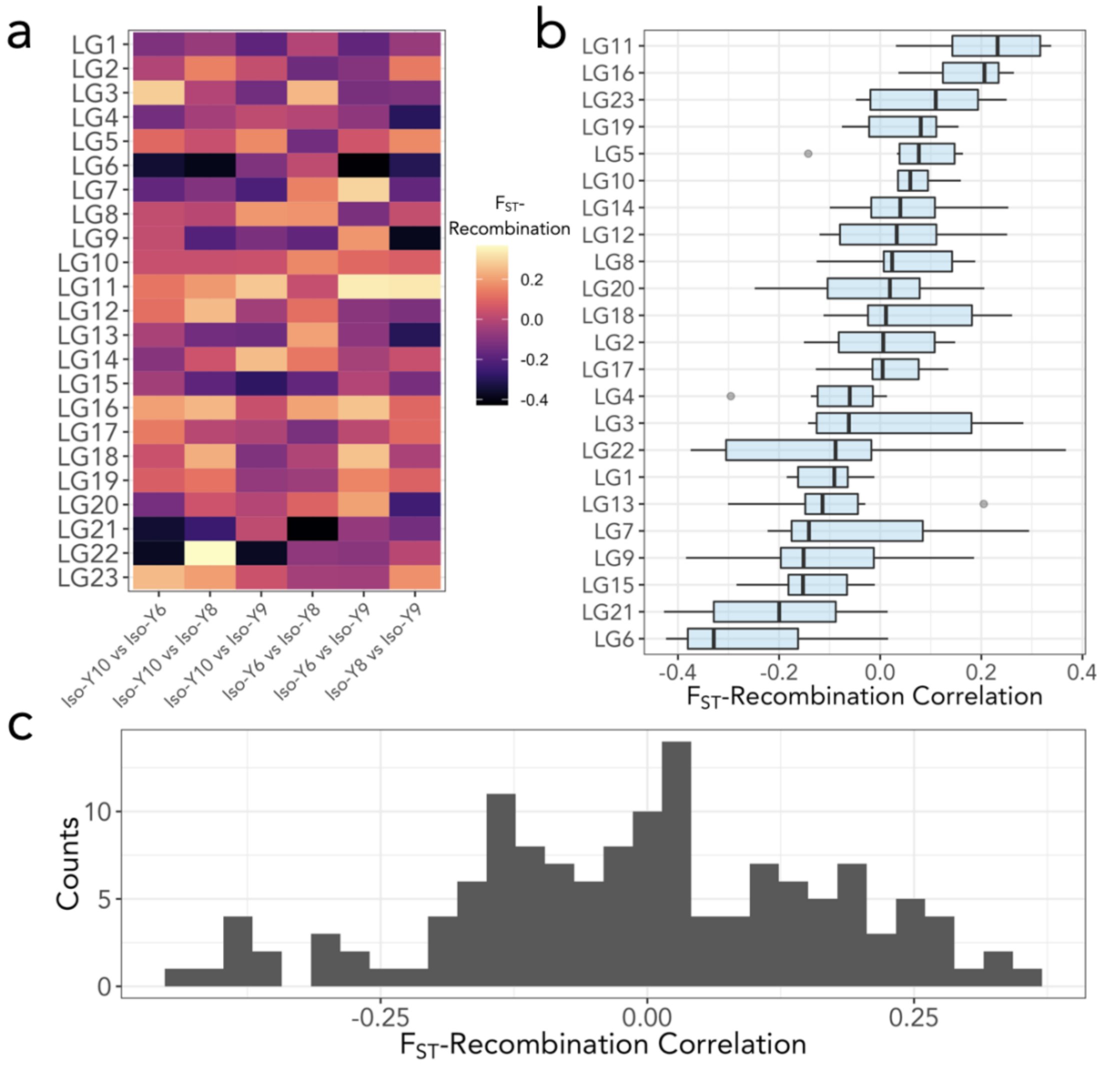

Supplementary Figure 6. Quantitative assessment of recombination and F_{ST} differentiation across the genome. Recombination data were extracted as outlined above in Supplementary Figure 5 [1]. a) Heatmap showing correlations (Kendall's tau) between the Iso-Y line pairwise F_{ST} estimates and recombination. Black represents a negative correlation (F_{ST} associated with low recombination) and white represents a positive correlation (F_{ST} associated with high recombination). b) Boxplots displaying the chromosome-wide correlation (Kendall's tau) between recombination and mean F_{ST} (in 100 Kbp windows) of the pairwise F_{ST} comparisons (n=6 independent comparisons). Data are presented following standard Tukey representations: minimum and maximum values of whiskers indicate 1.5 times the interquartile range (IQR), interquartile range is the length of the box, and median is a line through the box. c) Histogram of the distribution of genome-wide F_{ST} -recombination correlation. Source data are provided as a Source Data file.

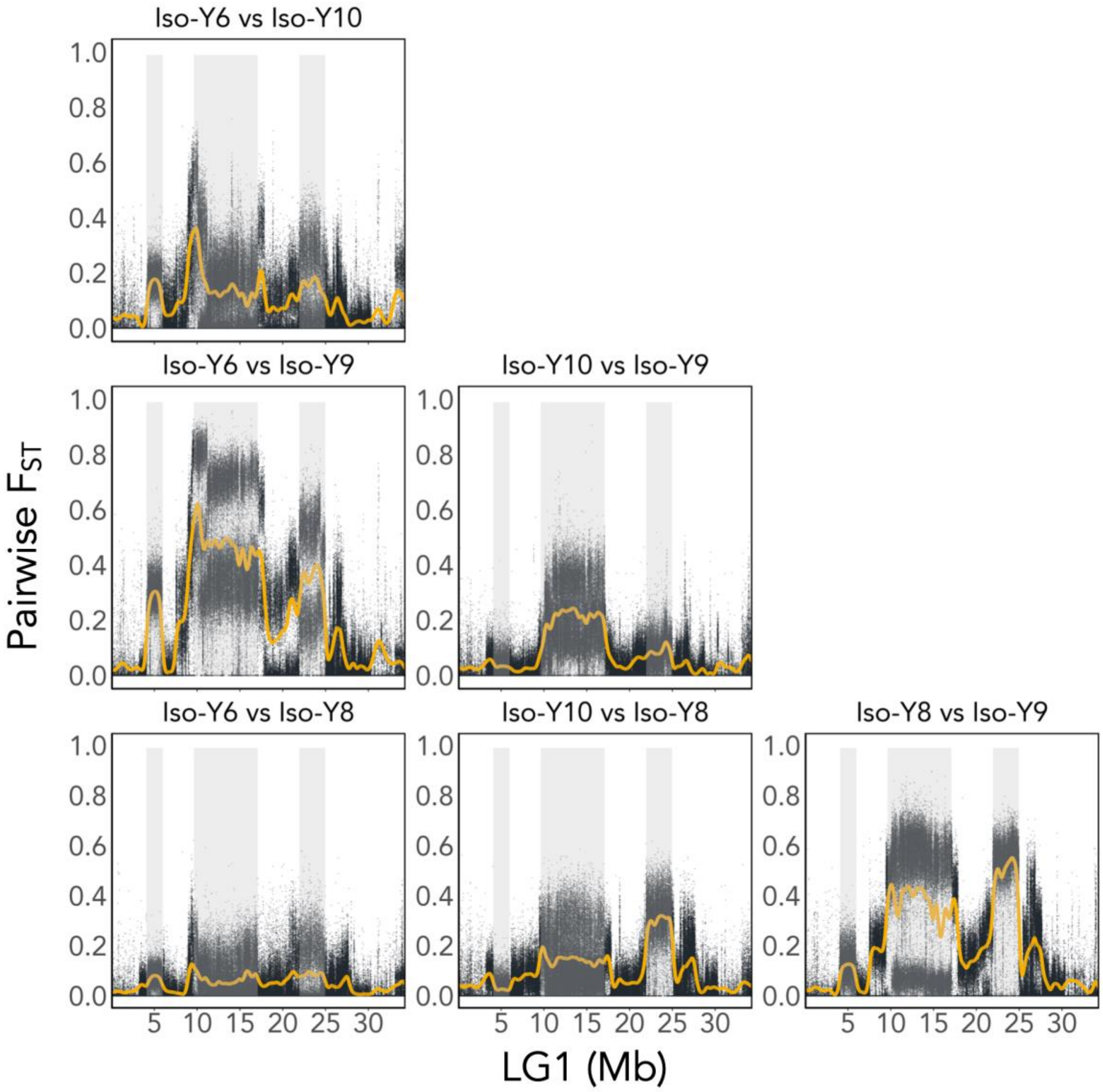

Supplementary Figure 7. LG1 Pairwise F_{ST} calculated between the four Iso-Y lines: Iso-Y6, Iso-Y8, Iso-Y9 and Iso-Y10. Yellow lines represent a smoothed spline of the data. Shaded areas represent the three regions identified by change point detection. Source data are provided as a Source Data file.

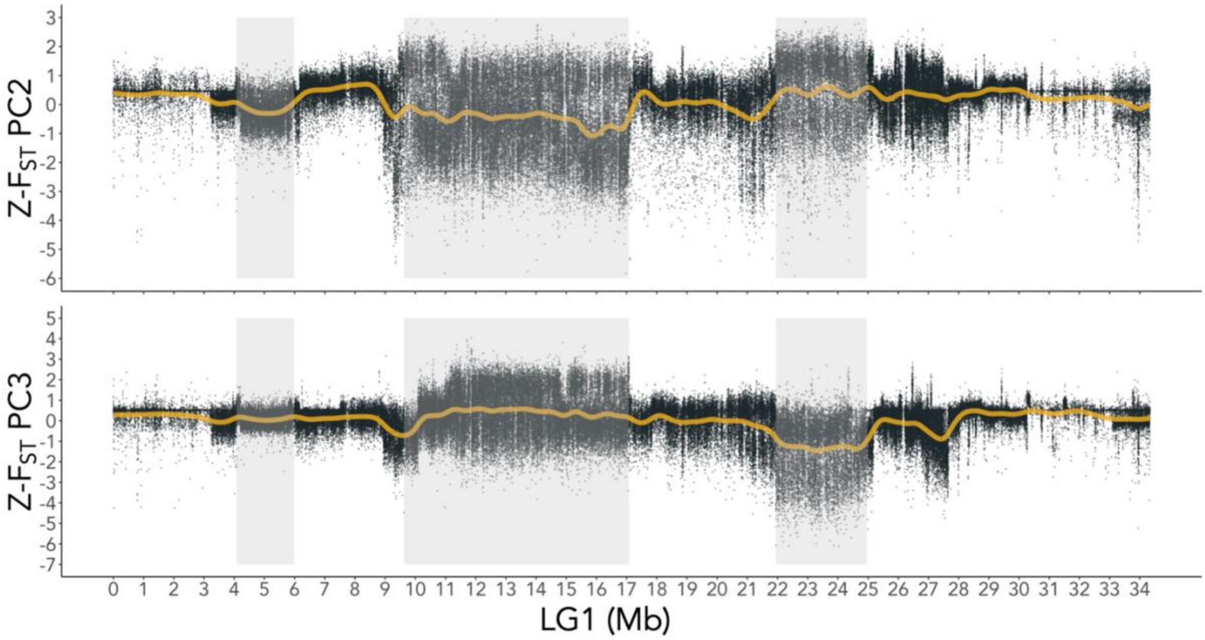

Supplementary Figure 8. Z-F_{ST} PC2 for LG1; yellow line represents a smoothed spline of the data. PC2 accounted for 17% of the total variance. PC3 accounted for 16% of the total variance. Shaded areas represent the three regions identified by change point detection. Source data are provided as a Source Data file.

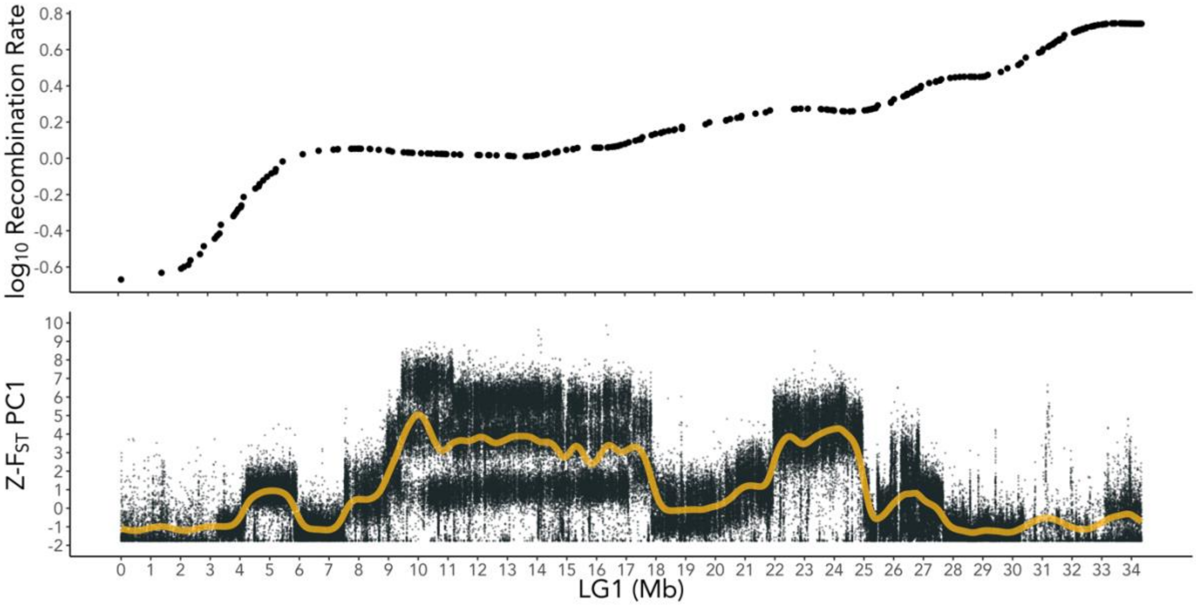

Supplementary Figure 9. Z-F_{ST} PC1 for LG1 and recombination rate. Smoothed recombination map were extracted as per the legend of Supplementary Figure 5 [1]. Recombination rates are presented as cM/Mb on a log10 scale. Source data are provided as a Source Data file.

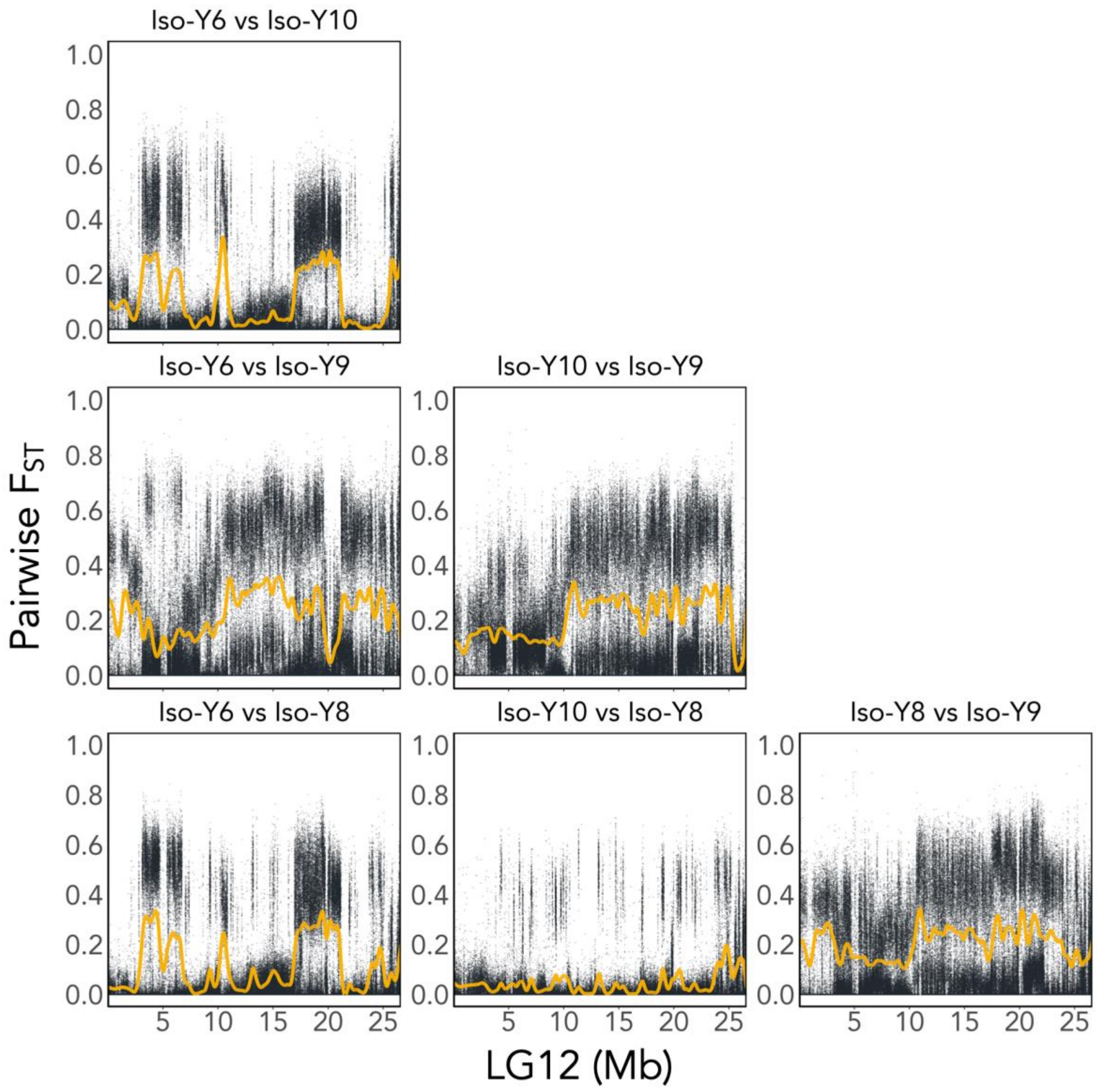

Supplementary Figure 10. LG12 Pairwise F_{ST} calculated between the four Iso-Y lines: Iso-Y6, Iso-Y8, Iso-Y9 and Iso-Y10. Yellow lines represent a smoothed spline of the data. No regions were identified by change point detection. Source data are provided as a Source Data file.

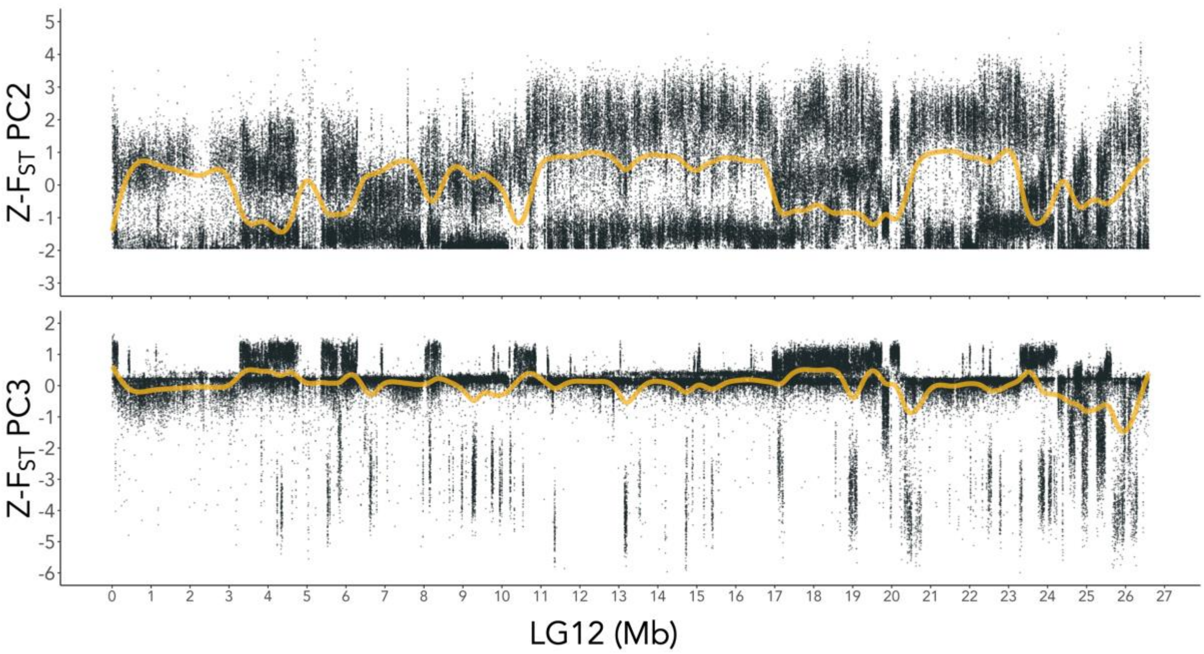

Supplementary Figure 11. Z-F_{ST} PC2 and Z-F_{ST} PC3 for LG12; yellow line represents a smoothed spline of the data. PC2 accounted for 30% of the total variance. PC3 accounted for 16% of the total variance. Source data are provided as a Source Data file.

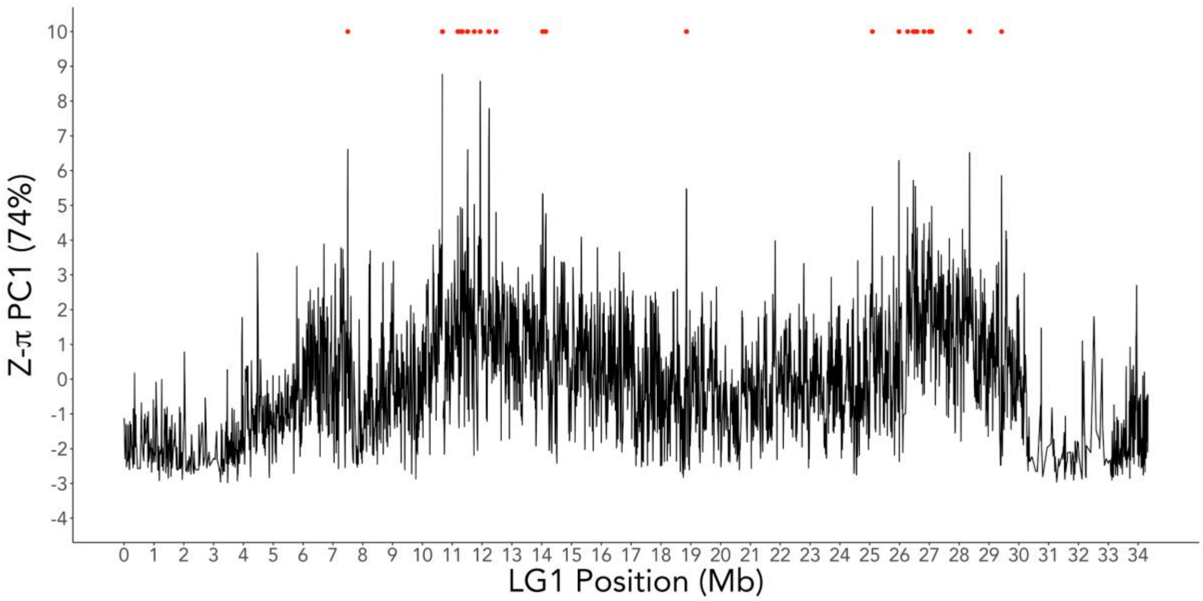

Supplementary Figure 12. Z-π-LG1 PC1 (74% of variance) along LG1, which represents the general diversity landscape of the chromosome shared by all Iso-Y lines (Supplementary Table 7). Red points mark 10kb windows with Z-π-LG1 PC1 above the upper 99% quantile (Z-π PC1 > 4.3). Source data are provided as a Source Data file.

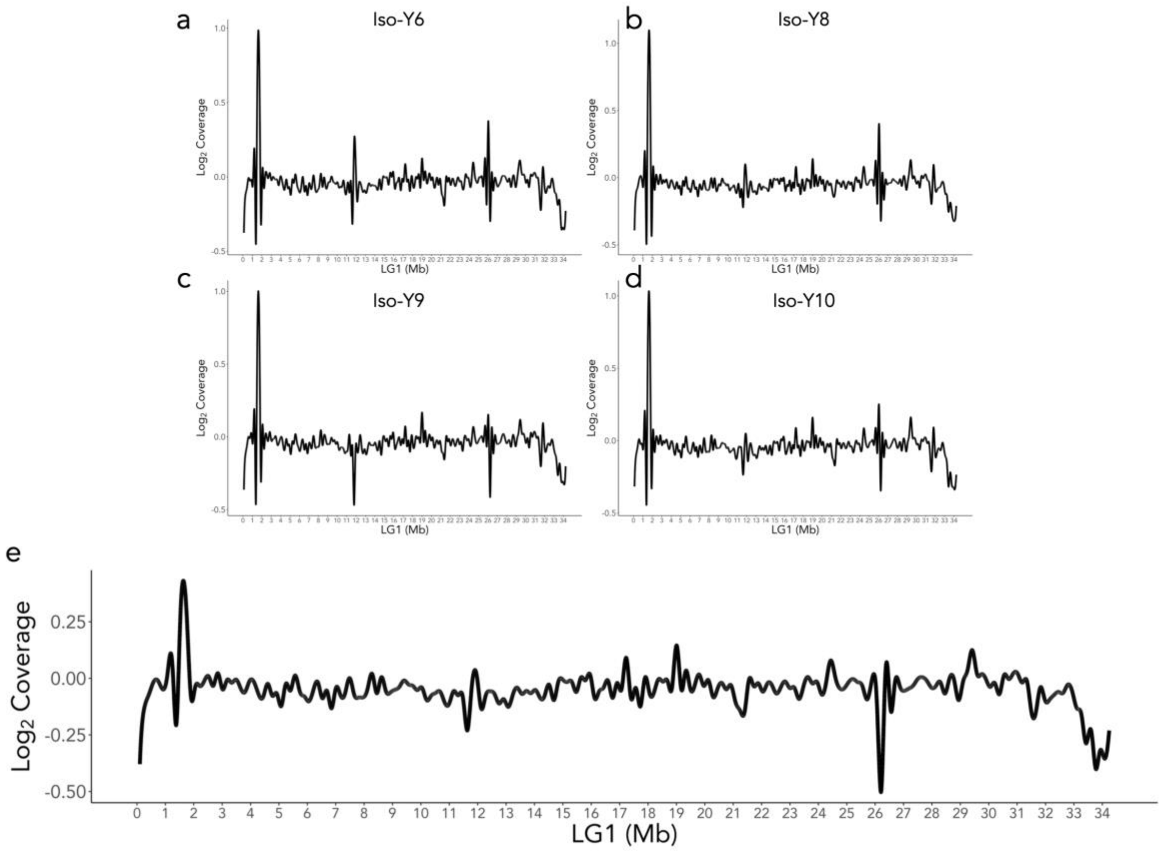

Supplementary Figure 13. Coverage calculated across LG1 for each of the four Iso-Y lines: a) Iso-Y6; b) Iso-Y8; c) Iso-Y9; d) Iso-Y10; e) averaged over the mean of all Iso-Y lines.

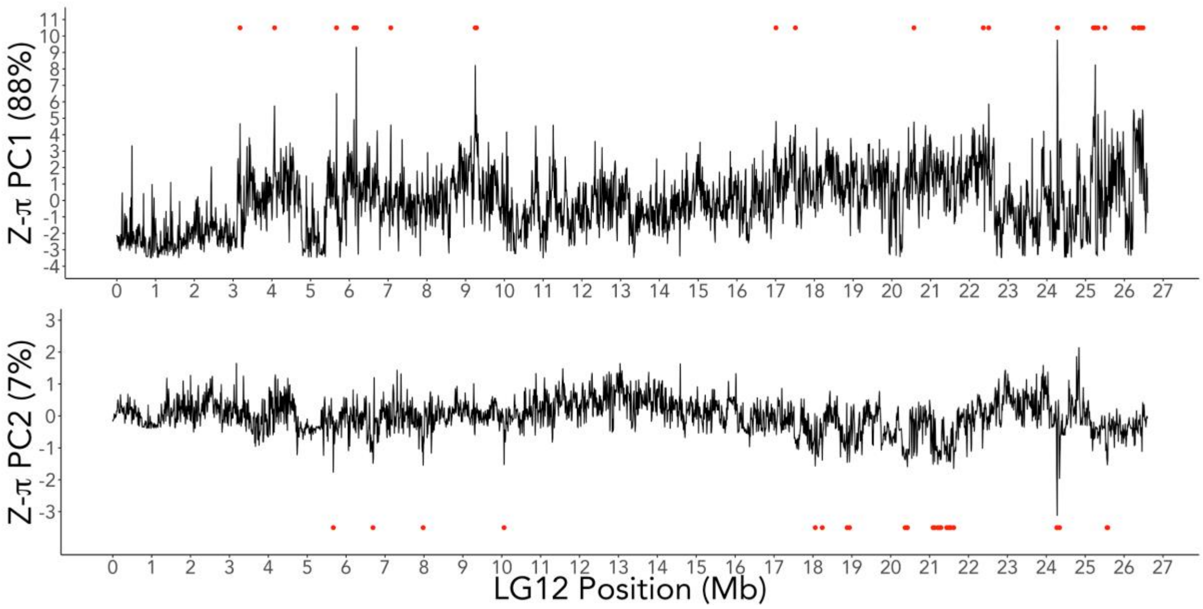

Supplementary Figure 14. Z-π PC1 (88% of variance) and Z-π PC2 (7% of variance) along LG12. PC1 represents the general diversity landscape of the chromosome shared by all Iso-Y lines (Supplementary Table 8). Red points mark 10kb windows with Z-π-LG1 PC1 and Z-π-LG1 PC2 above the upper 99% quantile (Z-π PC1 > 4.6; Z-π PC2 < -1.4). The highest scoring window on PC1 was found at 24.27 Mb. Iso-Y9 showed residual variance associated with PC2, where the highest scoring window on PC2 was found at 24.28 Mb. Source data are provided as a Source Data file.

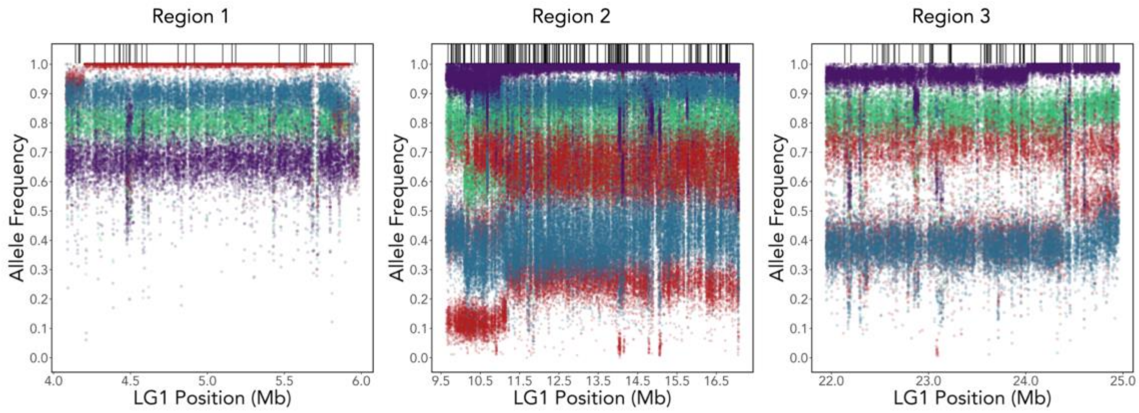

Supplementary Figure 15. LG1 polarised allele frequencies for Iso-Y6 (red), Iso-Y8 (blue), Iso-Y9 (purple) and Iso-Y10 (green) for the three identified regions of differentiation: a) Region 1, fixed in Iso-Y6 (coordinates: 4,079,988 - 5,984,584 bp); b) Region 2 (coordinates: 9,627,619 - 17,074,870 bp), fixed in Iso-Y9 and c) Region 3 (coordinates: 21,944,840 - 24,959,750 bp), fixed in Iso-Y9. Further detail of Region 2 and Region 3 can be found in Supplementary Figures 13 & 14. Source data are provided as a Source Data file.

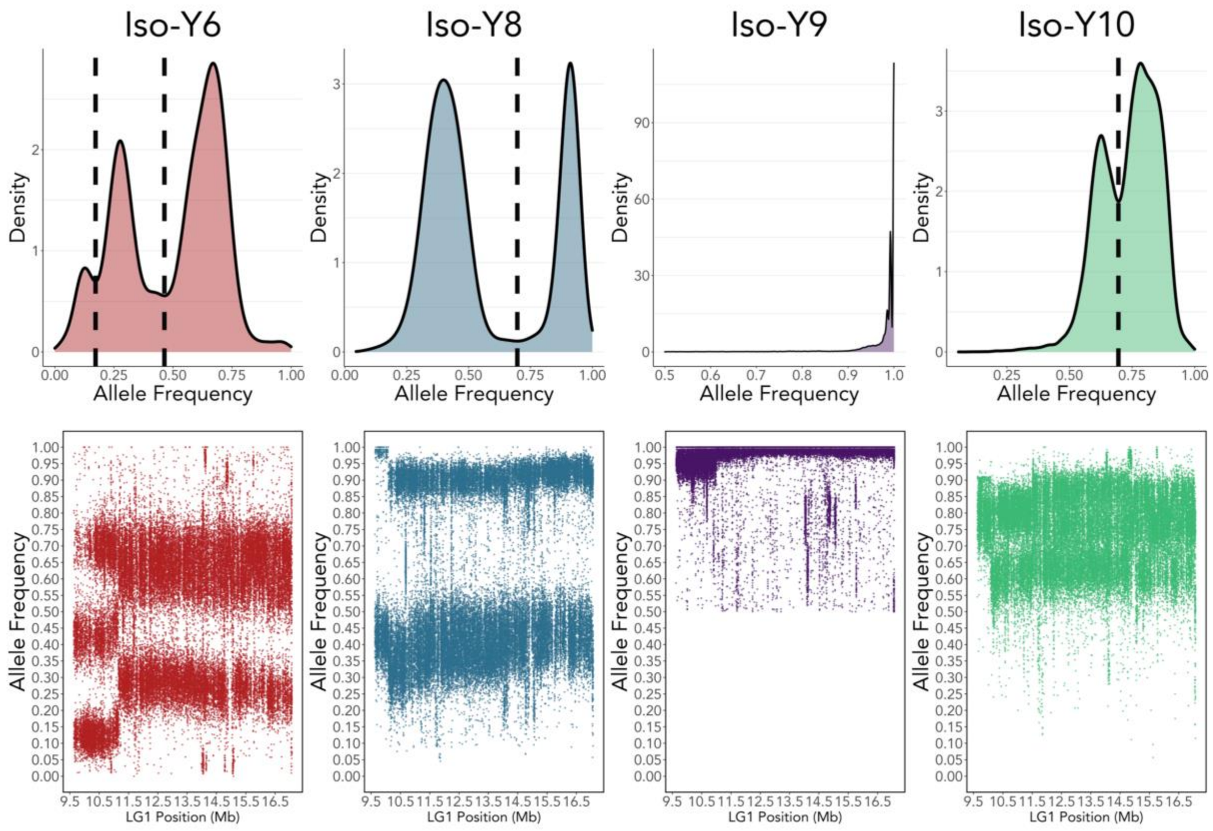

Supplementary Figure 16. Allele frequency (AF) density distributions for LG1 Region 2 (coordinates: 9,627,619 - 17,074,870 bp). Iso-Y6 (red) shows a trimodal distribution of AFs, with three distinct bands of segregating AFs in the first part of the region, and two distinct AF bands for the remainder of the region. Iso-Y8 (blue) and Iso-Y10 (green) both show bimodal AF distribution with two distinct bands of segregating AFs. Iso-Y9 (purple) shows fixation of the AFs. Source data are provided as a Source Data file.

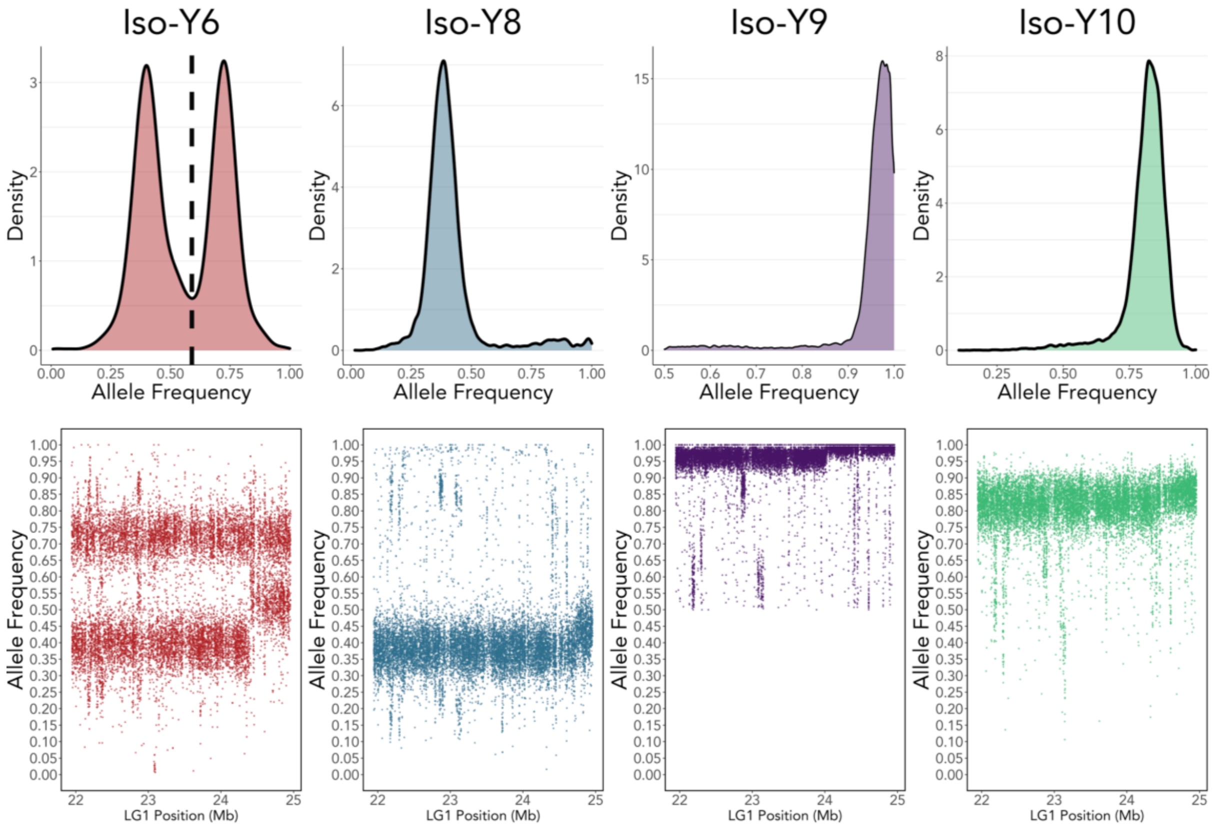

Supplementary Figure 17. Allele frequency (AF) density distributions for LG1 Region 3 (coordinates: 21,944,840 - 24,959,750 bp). Iso-Y6 (red) shows a bimodal distribution of AFs, with two distinct bands of segregating AFs. Iso-Y8 (blue), Iso-Y9 (purple) and Iso-Y10 (green) both show a single AF distribution with one band of segregating AFs. Iso-Y9 (purple) shows fixation of the AFs. Source data are provided as a Source Data file.

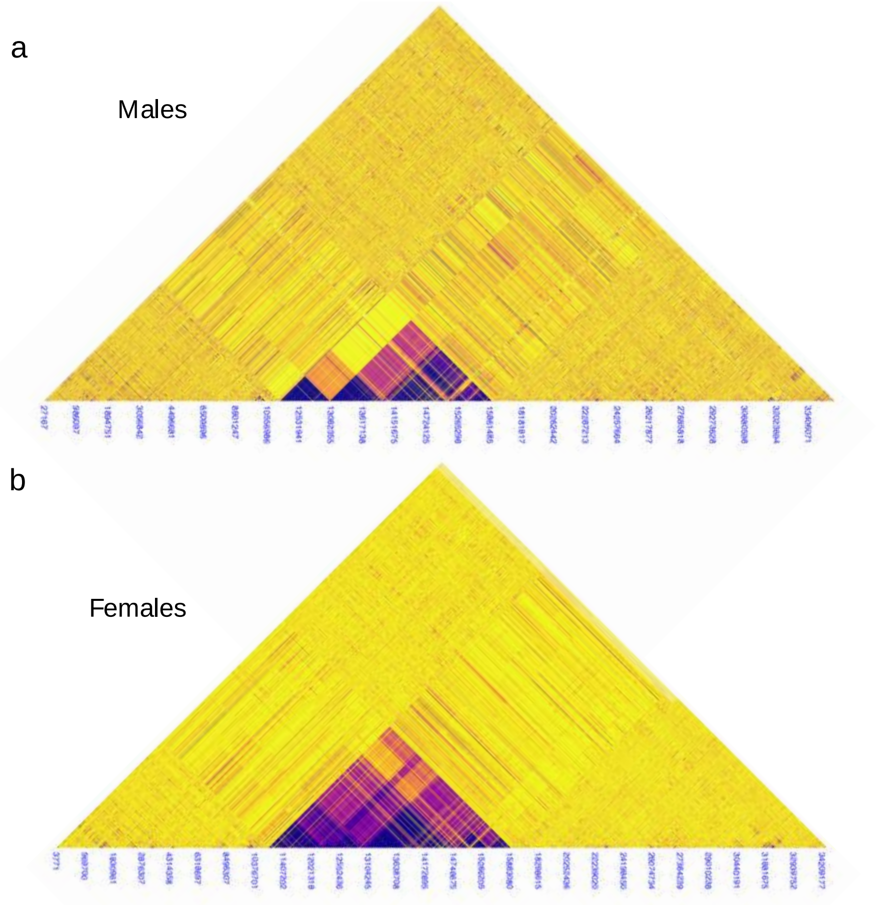

Supplementary Figure 18. Patterns of linkage disequilibrium (LD) in the natural data on LG1 for a) males (n=10) and b) females (n=16). Source data are provided as a Source Data file.

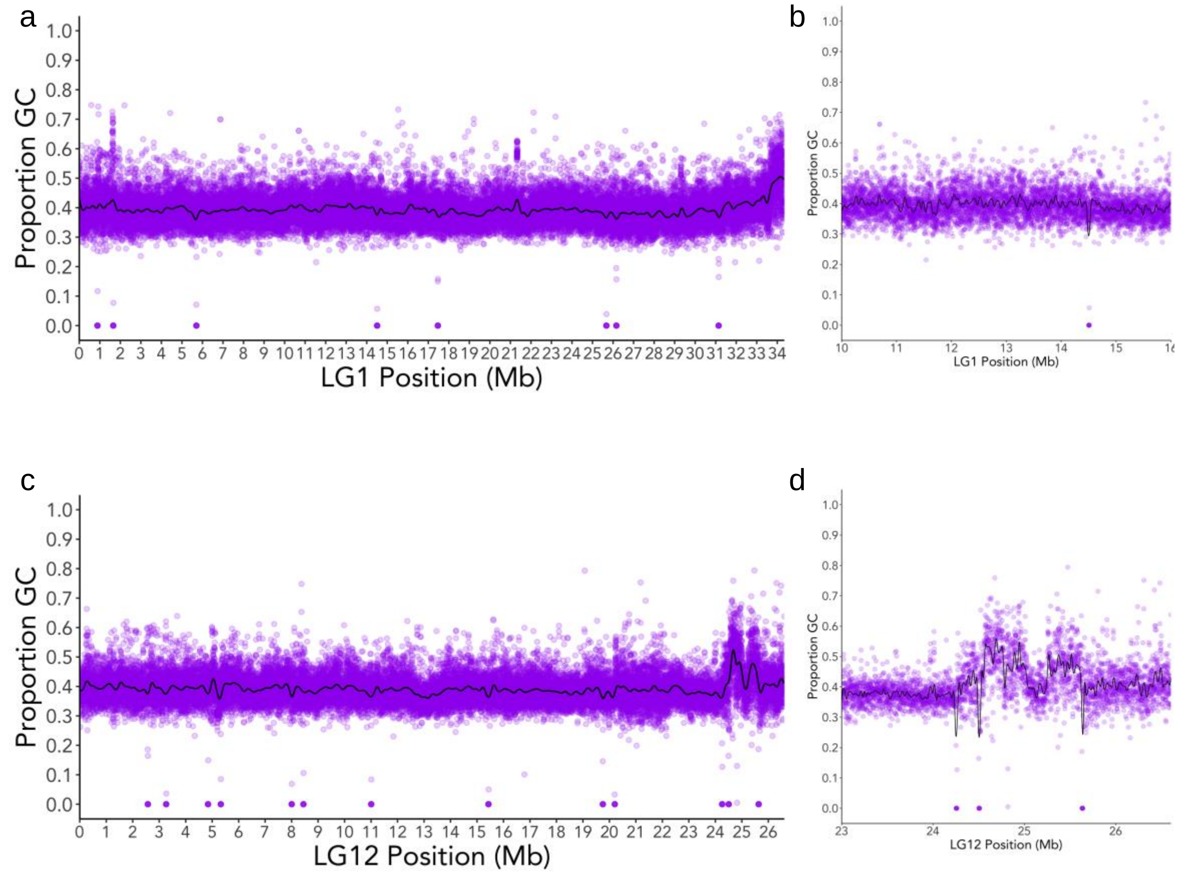

Supplementary Figure 19. GC content of LG1 and LG12 in 1kb windows. a) LG1 GC content; b) Zoom-in on LG1 region 2 GC content; c) LG12 GC content; d) Zoom-in on LG12 end-region GC content.

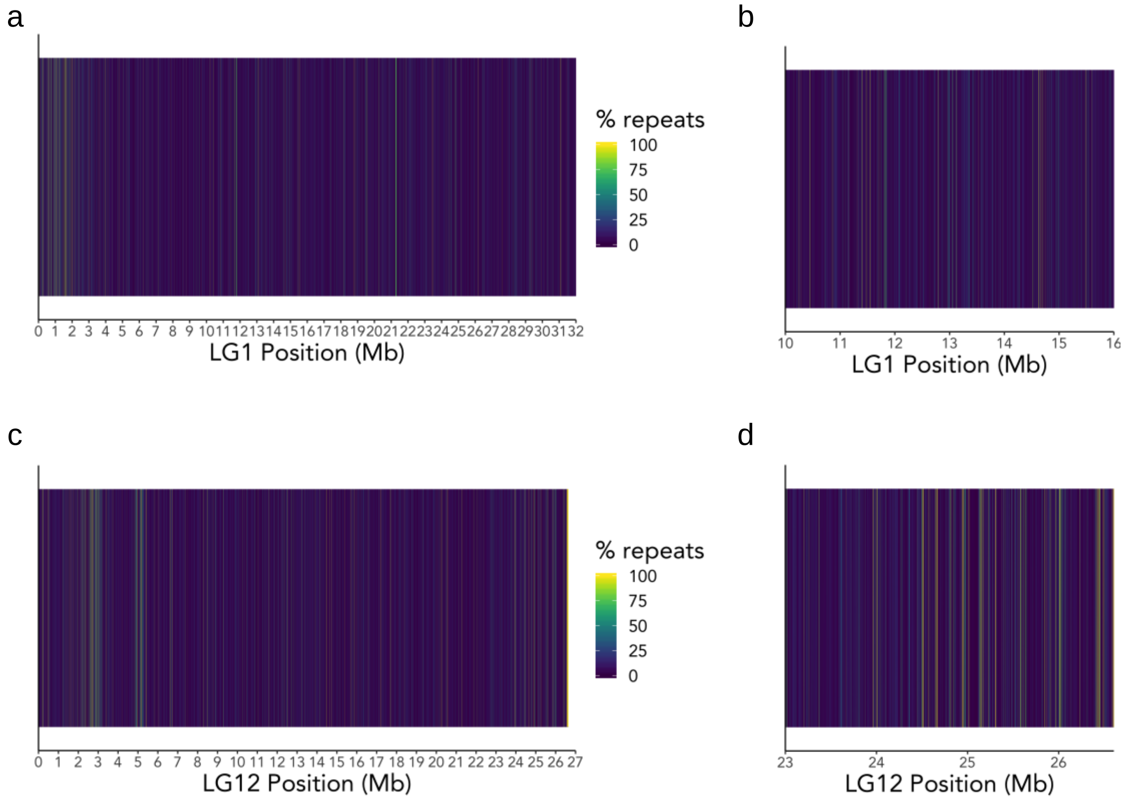

Supplementary Figure 20. Repeat content of LG1 and LG12 in 1kb windows. a) LG1 repeat content; b) Zoom-in on LG1 Region 2 repeat content; c) LG12 repeat content; d) Zoom-in on LG12 end-region repeat content.

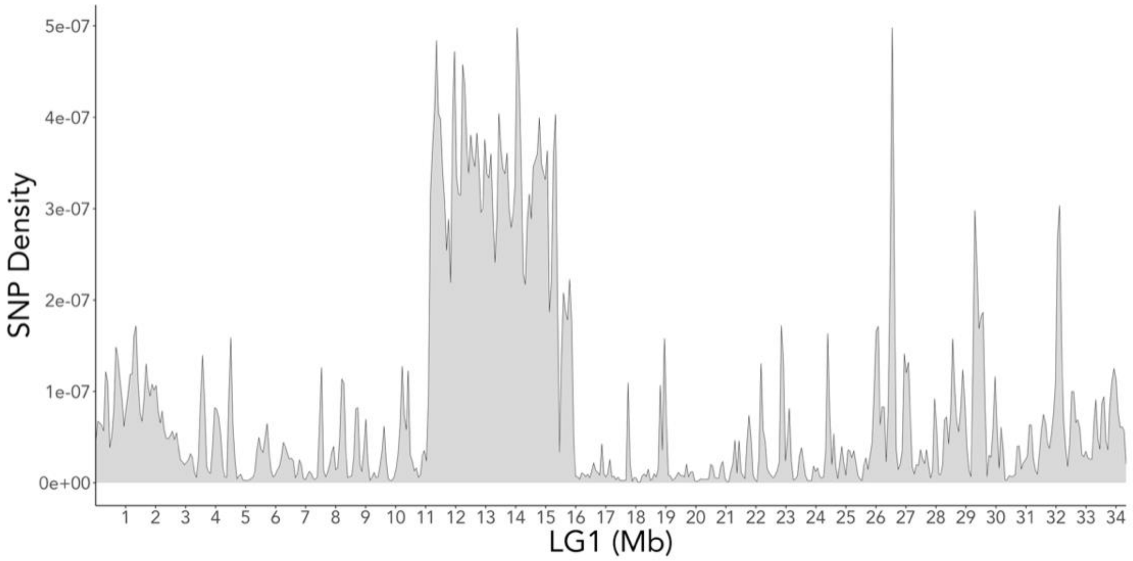

Supplementary Figure 21. LG1 SNP density in the natural data (n=26). Source data are provided as a Source Data file.

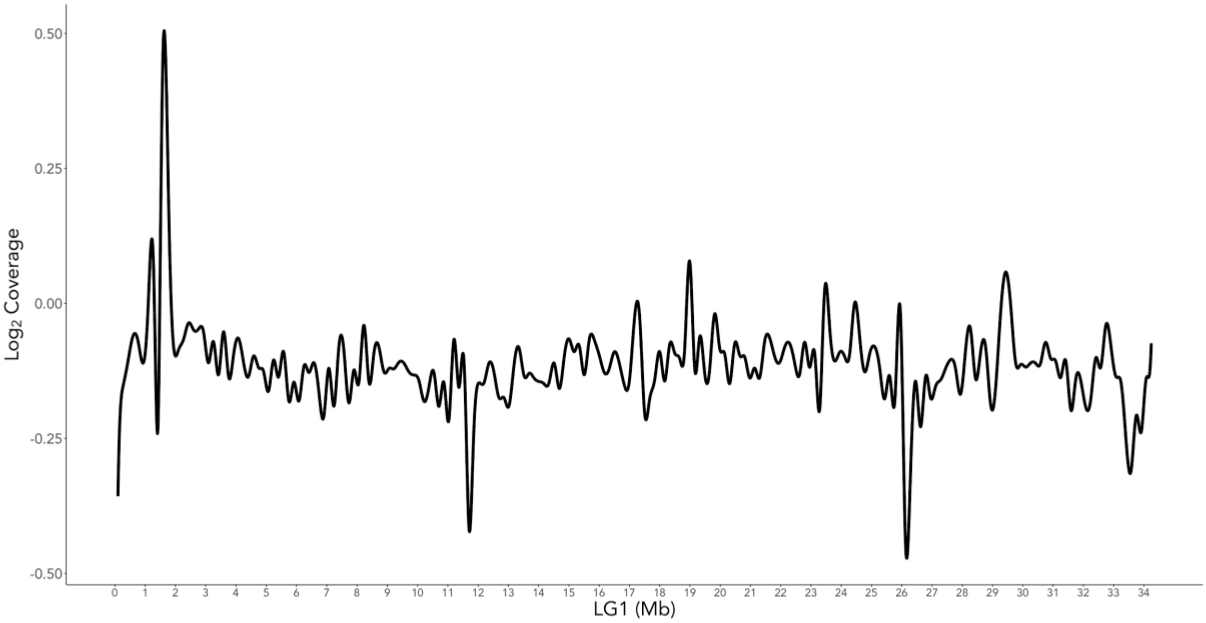

Supplementary Figure 22. Coverage calculated across LG1 for the natural data (n=26).

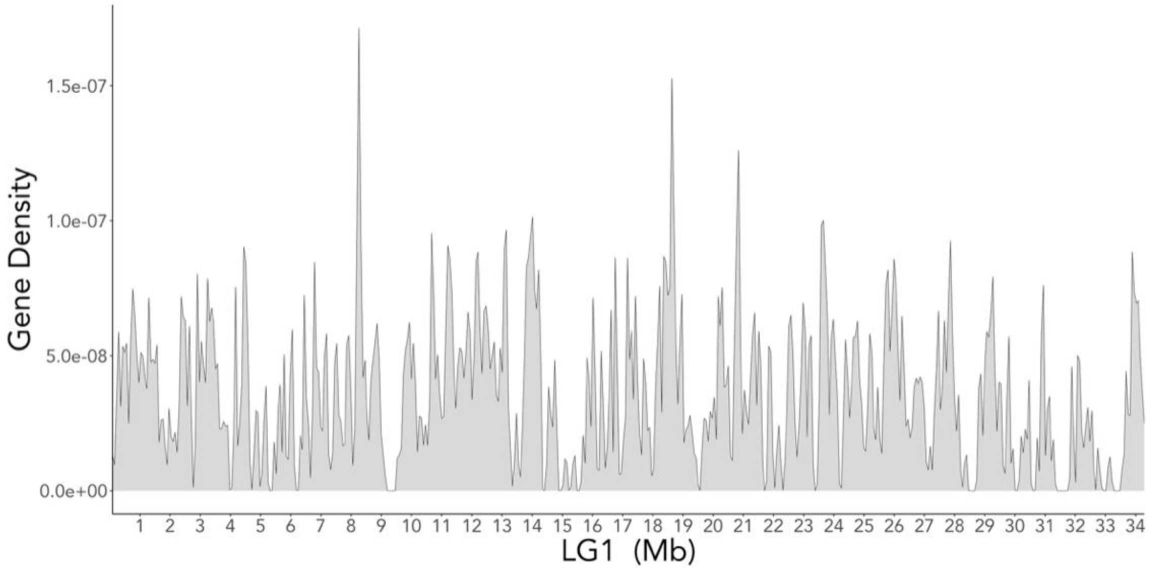

Supplementary Figure 23. Gene density of LG1 from the male guppy genome [4] (https://www.ebi.ac.uk/ena/browser/view/GCA_904066995).

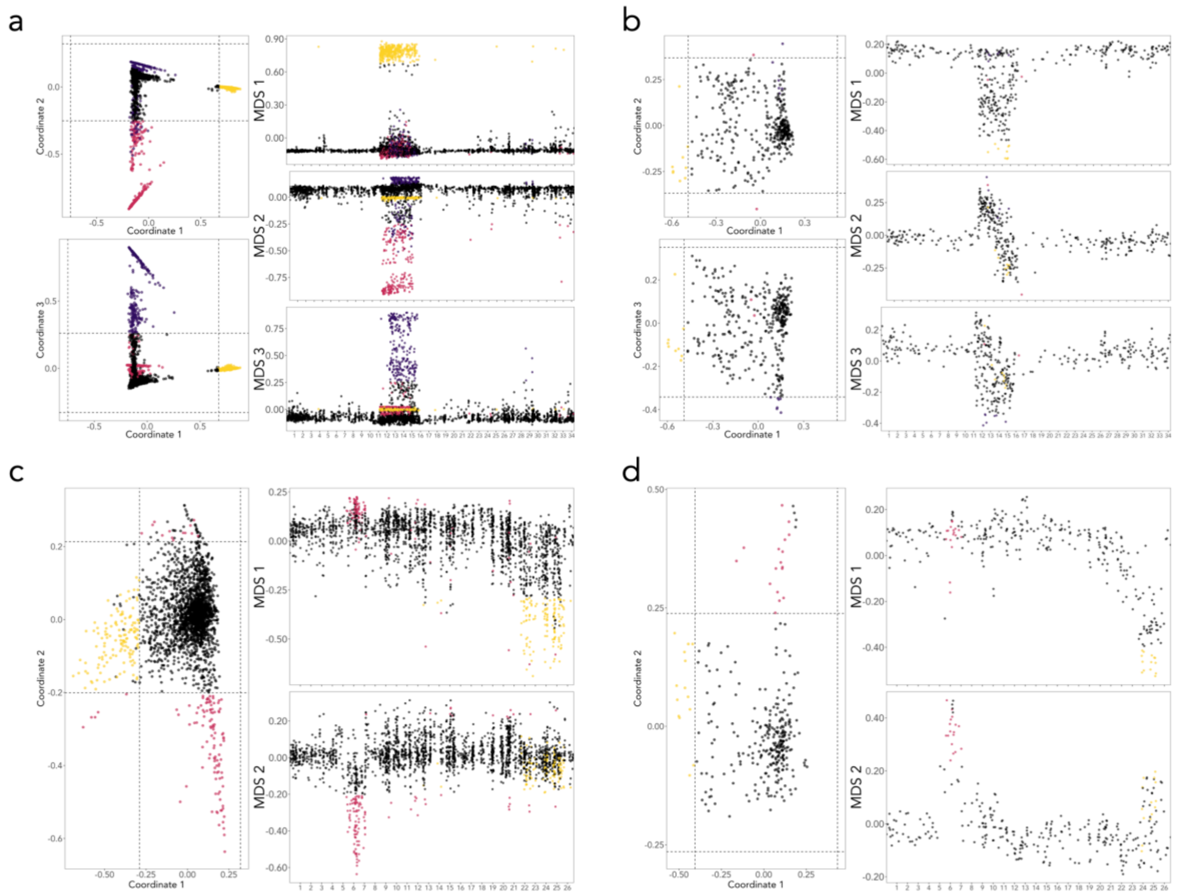

Supplementary Figure 24. *Lostruct* local PCA analysis for LG1 and LG12 in 10bp and 100bp windows. a) LG1 analysed in 10bp windows b) LG1 analysed in 100bp windows. LG1 plots both show the first 3 multidimensional scales (MDS: MD1: yellow; MDS2: pink; MDS3: purple). c) LG12 analysed in 10bp windows. d) LG12 analysed in 100bp windows. Although fewer windows are identified as outliers compared to 10bp windows, the same patterns of differentiation are apparent in both chromosomes.

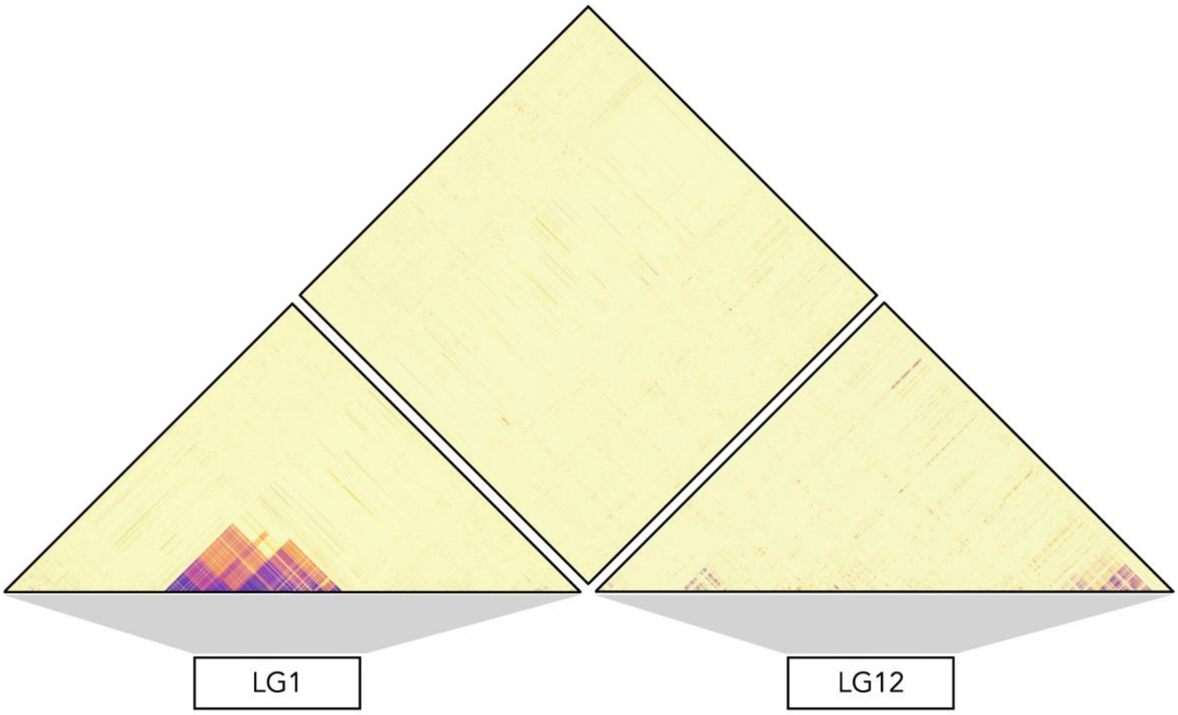

Supplementary Figure 25. Inter-chromosomal linkage between LG1 and LG12. Linkage information (R2) was calculated in Plink (--*inter-chr*), using only heterozygous sites at 5kb intervals.

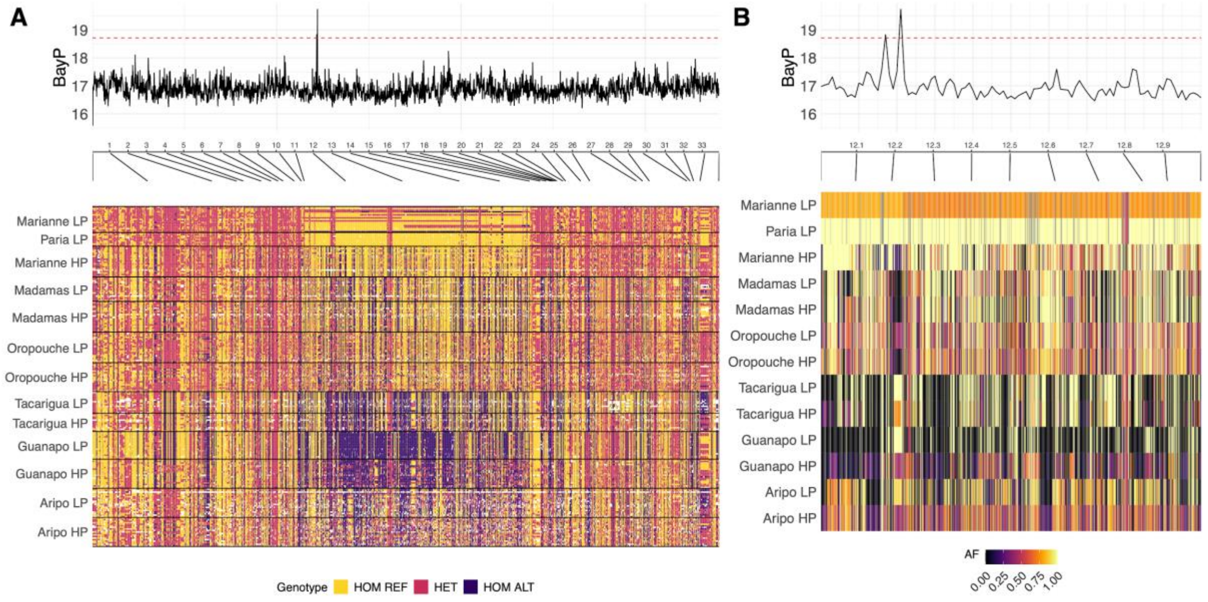

Supplementary Figure 26. Comparisons of LG1 among natural HP-LP populations and association with HP-LP adaptation. (a) BayPass association scores (BF) in 10kb windows along LG1 [5]. The first two rows show genotypes of individuals from the rivers considered in this study (Marianne LP and Paria LP). Remaining populations are considered in Whiting et al 2021. Genotypes are polarised to the major allele of Paria LP. This highlights a strong peak of HP-LP association between 12 and 13 Mb that overlaps with the sex-linked region of 12.1 to 13.2 Mb described in this study. This also demonstrates that the extended haplotype structure observed across LG1 Region 2-NP is not observed across all rivers in this extended dataset. (b) shows the region between 12 Mb and 13 Mb where the signal of HP-LP association is strongest. Each row highlights the allele frequencies (AF) for each SNP within this subset of the chromosome, again polarised to the major allele in Paria LP. The strongest signal of HP-LP association here (LG1:12210000-12220000), coincides with parallel allele frequency change among all rivers, except Madamas. In this region, all LP populations (except Madamas) exhibit an increased frequency, which is sometimes fixed, relative to their corresponding HP population for the major allele that is fixed in Paria.

Supplementary References

1. Whiting, J. R. *et al.* On the genetic architecture of rapidly adapting and convergent life history traits in guppies. *bioRxiv* 2021.03.18.435980 (2021) doi:10.1101/2021.03.18.435980
2. Lisachov, A. P., Zadesenets, K. S., Rubtsov, N. B. & Borodin, P. M. Sex chromosome synapsis and recombination in male guppies. *Zebrafish* **12**, 174–180 (2015)
3. Charlesworth, D. *et al.* Using GC content to compare recombination patterns on the sex chromosomes and autosomes of the guppy, Poecilia reticulata, and its close outgroup species. *Mol. Biol. Evol.* (2020) doi:10.1093/molbev/msaa187
4. Fraser, B. A., Whiting, J. R., Paris, J. R. & Bemm, F. *Guppy_genome: V1.0.0 - male guppy genome assembly*. (Zenodo, 2020). doi:10.5281/ZENODO.4020899
5. Whiting, J. R. *et al.* Drainage-structuring of ancestral variation and a common functional pathway shape limited genomic convergence in natural high- and low-predation guppies. *PLoS Genet.* **17**, e1009566 (2021)
