# Supplementary material for: A large and diverse autosomal haplotype is associated with sex-linked colour polymorphism in the guppy
Source: Nat Commun. 2022 Mar 9;13:1233. doi: 10.1038/s41467-022-28895-4 (PMC8907176; doi:10.1038/s41467-022-28895-4)
Supplement: Supplementary file 3 — Description of Additional Supplementary Files [file 41467_2022_28895_MOESM3_ESM.pdf]

Supplementary Data

Supplementary Data 1: Gene annotations for LG1 Region 1, Region 2 and Region 3

Supplementary Data 2: GO enrichment and KEGG mapping results

Supplementary Data 3: Gene annotations for LG12 MDS2 and MDS1

Supplementary Data 4: Results from SV analyses on both short-read and long-read data.

Supplementary Data 5: Sequencing and sample information for the natural WGS data
