# Supplementary material for: A large and diverse autosomal haplotype is associated with sex-linked colour polymorphism in the guppy
Source: Nat Commun. 2022 Mar 9;13:1233. doi: 10.1038/s41467-022-28895-4 (PMC8907176; doi:10.1038/s41467-022-28895-4)
Supplement: Supplementary file 9 — Reporting Summary [file 41467_2022_28895_MOESM9_ESM.pdf]

nature research

Corresponding author(s): Josephine R Paris

Last updated by author(s): Feb 1, 2022

Reporting Summary

Nature Research wishes to improve the reproducibility of the work that we publish. This form provides structure for consistency and transparency in reporting. For further information on Nature Research policies, see our Editorial Policies and the Editorial Policy Checklist.

Statistics

For all statistical analyses, confirm that the following items are present in the figure legend, table legend, main text, or Methods section.

n/a	Confirmed	
☐	☒	The exact sample size (*n*) for each experimental group/condition, given as a discrete number and unit of measurement
☐	☒	A statement on whether measurements were taken from distinct samples or whether the same sample was measured repeatedly
☐	☒	The statistical test(s) used AND whether they are one- or two-sided<br>*Only common tests should be described solely by name; describe more complex techniques in the Methods section.*
☒	☐	A description of all covariates tested
☐	☒	A description of any assumptions or corrections, such as tests of normality and adjustment for multiple comparisons
☐	☒	A full description of the statistical parameters including central tendency (e.g. means) or other basic estimates (e.g. regression coefficient) AND variation (e.g. standard deviation) or associated estimates of uncertainty (e.g. confidence intervals)
☐	☒	For null hypothesis testing, the test statistic (e.g. F, t, r) with confidence intervals, effect sizes, degrees of freedom and P value noted<br>*Give P values as exact values whenever suitable.*
☒	☐	For Bayesian analysis, information on the choice of priors and Markov chain Monte Carlo settings
☒	☐	For hierarchical and complex designs, identification of the appropriate level for tests and full reporting of outcomes
☒	☐	Estimates of effect sizes (e.g. Cohen's *d*, Pearson's *r*), indicating how they were calculated

Our web collection on statistics for biologists contains articles on many of the points above.

Software and code

Policy information about availability of computer code

Data collection	TPS series software (tpsUtil v1.81)was used to collect landmark data for fish morphometrics. No other software was used in the data collection process. Sequencing data were generated at the University of Exeter, through Illumina HiSeq and Pacbio technology. Please see Materials section and further detail below.
Data analysis	Colour measurement: tpsDig2 v2.31; tpsSuper v2.06; R v4.0.2; Colormesh v2.0; adegent v2.1.3; vegan v2.5-6. Sequencing data processing: cutadapt v1.13; bwa mem v0.7.17; samtools v1.9; qualimap v2.2.1; Freebayes v1.3.1; sambamba v0.7; parallel (20200322); vcftools v1.9; GATK v4.1.8.1; Beagle v5.0; Shapeit v2.r904; minimap2 v2.17; clusterProfiler v3.18.1; AnnotationHub v2.22.1; whatshap v0.18. Pool-seq and WGS analyses: poolfstat v1.2; stats v3.6.2; changepoint v2.2.2; biomaRt v2.44; bcftools v1.8; plink v1.9; LDheatmap v1.04; lostruct v0.9; PopGenome v2.7.5; BreakDancer v1.4.5; deeptools v3.3.1; smoove v0.2.5; SVtyper v0.7.0; Manta v1.6.0; sniffles v1.0.12; PBSV v2.3.0; IGV v2.4.8. R v4.0.1 was used for analyses throughout. Custom code is available at https://github.com/josieparis/guppy-colour-polymorphism and is archived under Zenodo with DOI: 10.5281/zenodo.5036660

For manuscripts utilizing custom algorithms or software that are central to the research but not yet described in published literature, software must be made available to editors and reviewers. We strongly encourage code deposition in a community repository (e.g. GitHub). See the Nature Research guidelines for submitting code & software for further information.

Data

Policy information about availability of data

All manuscripts must include a data availability statement. This statement should provide the following information, where applicable:

- Accession codes, unique identifiers, or web links for publicly available datasets
- A list of figures that have associated raw data
- A description of any restrictions on data availability

DNA sequencing data are available at the European Nucleotide Archive (ENA) under the Study Accession PRJEB36506: Pool-seq Iso-Y data (SAMEA6512722-SAMEA6512725); whole-genome sequencing data for Paria (SAMEA8750557-SAMEA8750565); long-read pacbio data for Iso-Y6 (SAMEA8795870-SAMEA8795872). Whole-genome sequencing data for Upper Marianne individuals are available from the ENA under the Study Accession PRJEB10680 (SAMEA3649957-SAMEA3649973). The male guppy reference genome can be accessed at the ENA under the Accession GCA_904066995 (https://www.ebi.ac.uk/ena/browser/view/GCA_904066995). The female guppy reference genome can be accessed at the ENA under Accession GCA_000633615.2 (https://www.ebi.ac.uk/ena/browser/view/GCA_000633615.2).

Field-specific reporting

Please select the one below that is the best fit for your research. If you are not sure, read the appropriate sections before making your selection.

☐ Life sciences ☐ Behavioural & social sciences ☒ Ecological, evolutionary & environmental sciences

For a reference copy of the document with all sections, see nature.com/documents/nr-reporting-summary-flat.pdf

Ecological, evolutionary & environmental sciences study design

All studies must disclose on these points even when the disclosure is negative.

Study description	In this study, we perform phenotyping and genotyping of individual Poecilia reticulata Iso-Y lines generated by a breeding design (described in full detail in the Methods). We also analyse sequencing data from 26 natural wild-caught Poecilia reticulata individuals.
Research sample	All samples used in this research are from the Trinidadian guppy (Poecilia reticulata).<br>Research samples originate from the Paria River, or nearby Upper Marianne River (Northern Mountain ranges of Trinidad). Males in this area are known to show strong Y-linkage of colour traits. Although the Paria and LP Marianne guppies are sampled from different sites, there is strong evidence that gene flow occurs between the populations occupying the upper reaches of these rivers due to regular flooding events. The Iso-Y lines were kindly provided by AE Houde, who established them by choosing male lineages from the Paria river in which colour pattern on the body was strongly Y-linked. The Iso-Y lines have been maintained at Florida State University since 2012.<br>For the Iso-Y lines, each line was founded by a single male drawn from the 'Houde' tributary of the Paria River (10.747404, -61.266287).<br>For the natural data, individuals were sampled from the Paria River (10.747404, -61.266287) and the Upper Marianne River (10.748574,-61.286622).
Sampling strategy	Wild-caught fish (natural data) were caught using butterfly nets and transported in sealed Nalgene bottles containing water with Stress Coat (API) to the nearby William Beebe Tropical Research Station, located in the lower Arima valley in the Northern Range, Trinidad. Sample sizes were determined based on availability of fish, taking a sufficient number for experimental design (target n=20) (as per Fumagalli M (2013) Assessing the Effect of Sequencing Depth and Sample Size in Population Genetics Inferences. PLOS ONE 8 (11): e79667. https://doi.org/10.1371/journal.pone.0079667) without over-disturbance of the local population, and within research budgets. The sample size of the Iso-Y lines for Pool-seq was determined following the guidance of Schlötterer, C., Tobler, R., Kofler, R. et al. Sequencing pools of individuals — mining genome-wide polymorphism data without big funding. Nat Rev Genet 15, 749–763 (2014). https://doi.org/10.1038/nrg3803.
Data collection	Data were collected in the field by recording sex and number of fish using a pen and paper. BAF performed the field collection for the Upper Marianne individuals. FHR performed the field collection for the Paria individuals. Iso-Y individuals were originally collected by AE Houde, and have been maintained at Florida State University since 2012. DNA extraction and laboratory work was performed by JRP, PJP and MvdZ. MJD performed the colour phenotyping data collection using TPS series software. Sequencing data were collected on the University of Exeter's High Performance Cluster (HPC) ISCA for analysis.
Timing and spatial scale	Sampling for the wild-caught individuals from Upper Marianne was conducted in September of 2013 by BAF. Sampling for the wild-caught individuals from Paria was conducted in May of 2016 by FHR. Spatial scale of Upper Marianne sampling was <1km. Spatial scale of Paria sampling was 0.33km. Access to the sites is dependent on local weather (e.g. flooding), security and health and safety. Sequencing was conducted in 2018-2019. Analysis was performed 2019-2021.
Data exclusions	No data were excluded
Reproducibility	All code and scripts were bug tested by JRP, JRW and BAF, including replication of findings. There is no experimental animal model part of this research.

Randomization	This study does not involve human participants. A random sample of fish were taken from the wild. We randomly sampled Iso-Y males used for sequencing.
Blinding	Not relevant to this study - this study is not a clinical trial.

Did the study involve field work? ☒ Yes ☐ No

Field work, collection and transport

Field conditions	For the natural data, fieldwork was conducted in September of 2013 and May of 2016 under good weather conditions and prior to the rainy season. There was no rain on the day of sampling at either Upper Marianne nor Paria sampling locations. Temperature for Upper Marianne was 27-30 degrees C. Temperature for Paria sampling was 25-26 degrees C. Fish were transported from the field to the William Beebe Tropical Research Station.
Location	For the Iso-Y lines, each line was founded by a single male drawn from the 'Houde' tributary of the Paria River (10.747404, -61.266287).<br>For the natural data, individuals were sampled from the Paria River in 2016 (10.747404, -61.266287) and the Upper Marianne River in 2013 (10.748574,-61.286622).<br>Water depth varied between 20-120cm
Access & import/export	Care is taken not to damage habitat by walking on established paths to the collecting site. Researchers walk in the stream to avoid trampling the vegetation on the stream banks. Butterfly nets are used to gently collect only the species and specific specimens required for this study. Fish were collected and exported from Trinidad with permits granted from the Aquaculture Unit, of Fisheries Division, Ministry of Agriculture, Land and Fisheries. Permits for Paria were issued in March 2016 (valid for the year of 2016). Permits for Upper Marianne were issued in September 2013 (valid for the year of 2013). If necessary, copies of these permits can be made available on request.
Disturbance	Poecilia reticulata are non-threatened, abundant species. therefore taking small samples will not cause great disturbance

Reporting for specific materials, systems and methods

We require information from authors about some types of materials, experimental systems and methods used in many studies. Here, indicate whether each material, system or method listed is relevant to your study. If you are not sure if a list item applies to your research, read the appropriate section before selecting a response.

Materials & experimental systems

n/a	Involved in the study	
☒	☐	Antibodies
☒	☐	Eukaryotic cell lines
☒	☐	Palaeontology and archaeology
☐	☒	Animals and other organisms
☒	☐	Human research participants
☒	☐	Clinical data
☒	☐	Dual use research of concern

Methods

n/a	Involved in the study	
☒	☐	ChIP-seq
☒	☐	Flow cytometry
☒	☐	MRI-based neuroimaging

Animals and other organisms

Policy information about studies involving animals; ARRIVE guidelines recommended for reporting animal research

Laboratory animals	No laboratory animals were used in this study.
Wild animals	All fish used are species Poecilia reticulata. Fish were all adults > 3 months of age.<br>For the Iso-Y line colour analysis: Iso-Y6 n = 41; Iso-Y8 n= 48; Iso-Y9 n= 42; Iso-Y10 n= 42 (Total = 173). All mature males.<br>For the Iso-Y line sequencing analysis: n per Iso-Y line = 48 (Total = 192). All mature males.<br>For the natural data analysis: n females = 16, n males = 10 (Total = 26). All mature adults.<br>For DNA extraction, fish were humanely killed under USA licensed procedures (chilling followed by decapitation), as approved by the relevant ethics boards (see below).
Field-collected samples	Not relevant to this study.
Ethics oversight	The research presented was described in Animal Research Protocol No. 1442 approved on 29 October 2014 and Protocol No. 1740 approved on 16 October 2017, by the Animal Care and Use Committee of Florida State University.

Note that full information on the approval of the study protocol must also be provided in the manuscript.
